# Non-photoreceptor Expression of *Tulp1* May Contribute to Extensive Retinal Degeneration in *Tulp1-/-* Mice

**DOI:** 10.3389/fnins.2020.00656

**Published:** 2020-06-23

**Authors:** Arpad Palfi, Adlet Yesmambetov, Pete Humphries, Karsten Hokamp, G. Jane Farrar

**Affiliations:** Department of Genetics, Trinity College Dublin, Dublin, Ireland

**Keywords:** retina, degeneration, mouse model, inherited, *Tulp1*, disease, blindness, eye

## Abstract

Mutations in tubby like protein 1 gene (*TULP1*) are causative of early-onset recessive inherited retinal degenerations (IRDs); similarly, the *Tulp1-/-* mouse is also characterized by a rapid IRD. *Tulp1* mRNA and protein expression was analyzed in wild type mouse retinas and expression data sets (NCBI) during early postnatal development. Comparative histology was undertaken in *Tulp1-/-*, rhodopsin-/- (*Rho-/-*) and retinal degeneration slow-/- (*Rds-/-*) mouse retinas. Bioinformatic analysis of predicted TULP1 interactors and IRD genes was performed. Peak expression of *Tulp1* in healthy mouse retinas was detected at p8; of note, TULP1 was detected in both the outer and inner retina. Bioinformatic analysis indicated *Tulp1* expression in retinal progenitor, photoreceptor and non-photoreceptor cells. While common features of photoreceptor degeneration were detected in *Tulp1-/-*, *Rho-/-*, and *Rds-/-* retinas, other alterations in bipolar, amacrine and ganglion cells were specific to *Tulp1-/-* mice. Additionally, predicted TULP1 interactors differed in various retinal cell types and new functions for TULP1 were suggested. A pilot bioinformatic analysis indicated that in a similar fashion to *Tulp1*, many other IRD genes were expressed in both inner and outer retinal cells at p4–p7. Our data indicate that expression of *Tulp1* extends to multiple retinal cell types; lack of TULP1 may lead to primary degeneration not only of photoreceptor but also non-photoreceptor cells. Predicted interactors suggest widespread retinal functions for TULP1. Early and widespread expression of TULP1 and some other IRD genes in both the inner and outer retina highlights potential hurdles in the development of treatments for these IRDs.

## Introduction

Mutation in over 300 genes have been identified as causative of inherited retinal degenerations (IRDs) (Duncan et al., 2018; RetNet - Retinal Information Network, 2019) affecting 1 in 3000, or more than 2 million people worldwide (Sahel et al., 2015; Farrar et al., 2017). The impact of IRDs on vision ranges from mild impairment to complete blindness; IRDs represent a significant economic and social burden including devastating psychological and quality-of-life impacts on patients and families (Smith et al., 2015; Retina International, 2019).

Mutations in tubby like protein 1 gene (*TULP1*) are causative of rare, early onset, severe forms of autosomal recessive retinal degeneration, usually diagnosed as Leber congenital amaurosis 15 (LCA15) or retinitis pigmentosa 14 (RP14) (RetNet - Retinal Information Network, 2019). TULP1 is highly specific to photoreceptors in the mouse retina (Hagstrom et al., 1999; Xi et al., 2005; Grossman et al., 2009), although lower levels of TULP1 have been detected in ganglion and progenitor cells in human retinas (Milam et al., 2000). To enhance understanding of *TULP1-*linked IRDs, *Tulp1-/-* mice were generated (Hagstrom et al., 1999; Ikeda et al., 2000). *Tulp1-/-* mice exhibit an early and severe retinal degeneration akin to the human condition; shortening of photoreceptor segments and swollen extruded mitochondria by postnatal day (p)14 (Ikeda et al., 2000); abnormal ribbon synaptic architecture by p13–p16 (Grossman et al., 2009); shortening of bipolar cell dendrites with less branching and compromised b-wave electroretinogram (ERG) by p16 (Grossman et al., 2009); reduced rod and cone ERGs by week 4 (Hagstrom et al., 1999; Ikeda et al., 2000); photoreceptor apoptosis from p18 (Ikeda et al., 2000) resulting in complete loss of the outer nuclear layer (ONL) by week 20 (Hagstrom et al., 1999; Ikeda et al., 2000).

The function of TULP1 has not been clearly established. In photoreceptors, TULP1 is colocalized with f-actin in the inner segments (Xi et al., 2005), where it may be engaged in trafficking of proteins such as rhodopsin (RHO) and cone opsins between the inner and outer segments (Grossman et al., 2011; Hagstrom et al., 2012). TULP1 is also required for normal development of photoreceptor synapses and survival of photoreceptor cells (Grossman et al., 2009). TULP1 interacts with the synaptic ribbon protein (RIBEYE) and mediates localization of the endocytic machinery at the periactive zone of photoreceptor synapses (Wahl et al., 2016). Direct interaction between dynamin-1 (DNM1) and TULP1 highlights the role of TULP1 in synaptic vesicular transport (Xi et al., 2007) (Grossman et al., 2013). TULP1 also interacts with the microtubule associated protein 1b (MAP1B) (Grossman et al., 2014). Additionally, TULP1 is a ligand for MER proto-oncogene tyrosine kinase (MERTK) and facilitates phagocytosis in retinal pigment epithelium (RPE) cells (Caberoy et al., 2010).

As TULP1 has been detected in retinal ganglion and progenitor cells in human retinas (Milam et al., 2000), we hypothesized that likewise, TULP1 may not be exclusively specific to photoreceptors in mice. The *Tulp1-/-* retina may represent a model where aspects of primary photoreceptor and non-photoreceptor degenerations could be studied. Therefore, we explored non-photoreceptor expression of *Tulp1* in the murine retina and assessed the potential impact of lack of TULP1 in non-photoreceptor cells in *Tulp1-/-* mice. We also considered, whether TULP1 may be expressed in the early post-natal retina of mice, which may contribute to the severe retinal degeneration observed in *Tulp1-/-* mice.

The p5–p30 period was selected for analysis, a timeframe which overlaps with a substantial part of postnatal development of the mouse retina and precedes photoreceptor degeneration in *Tulp1-/-* mice. Immunocytochemistry and bioinformatic analysis indicated *Tulp1* expression in both the outer and inner retina in wild type (wt) mice. Using various cellular markers, we evaluated the architecture of *Tulp1-/-* retinas compared to retinas from *Rho-/-* (Humphries et al., 1997) and retinal degeneration slow (*Rds-/-*) (Sanyal et al., 1980) mice, where retinal degeneration is believed to originate from photoreceptors. Significant differences between the inner retina of *Tulp1-/-* versus the *Rho-/-* and *Rds-/-* retinas were identified. We suggest that these may reflect the effects of expression of *Tulp1* in multiple non-photoreceptor cells. Bioinformatic analysis of expression of the predicted TULP1 interactome suggests cell type-specific utility of TULP1 in the retina. Additionally, bioinformatic analysis indicated that a similar profile of expression in both the outer and inner retina is observed for a number of other IRD genes at p4–p7.

## Materials and Methods

### Animals

The following transgenic mice were used in this study; *Tulp1-/-* (B6.129X1-Tulp1tm1Pjn/Pjn; The Jackson Laboratory) (Ikeda et al., 2000), *Rho-/-* (Humphries et al., 1997), and *Rds-/-* (Sanyal et al., 1980). The background strain of these mice was C57BL/6J (except for *Rds-/-*, which was O20/A; this line does not exist anymore); wt C57BL/6J mice were used as controls. Mice were maintained under specific pathogen free (SPF) housing conditions; both sexes were used for experiments. Animal welfare complied with the Directive 2010/63/EU; Protection of Animals Used for Scientific Purposes, Regulations 2012 (S.I. No. 543 of 2012) and the Association for Research in Vision and Ophthalmology (ARVO) Statement for the Use of Animals in Ophthalmic and Vision Research.

### Expression Analysis of GEO Data Sets

*Tulp1*, TULP1 interactor and IRD mRNA expression was analyzed in retinal cell type-specific mouse data sets (GSE71462, GSE19304, GSE127771, GSE80232, GSE81903, GSE33088, GSE86199, GSE115404, GSE90652) downloaded from the Gene Expression Omnibus (GEO) resource. Depending on the microarray platform used, raw CEL files were processed either with GCRMA and Limma (GSE59201) or with the oligo package from Bioconductor (GSE33088) to derive normalized expression values. GSE19304, for which raw expression values were provided, was normalized with the tmm method (Robinson and Oshlack, 2010) in R. Bulk RNA sequencing data were transformed from counts or FPKM/RPKM (fragments/reads per kilobasepairs per million) values to TPM (transcripts per million) values using Perl scripts^1^. Technical and biological replicates in each data set were averaged. Normalization between data sets was carried out by dividing each data point by the average of the housekeeping genes; *Actb*, *B2m*, *Gapdh*, *Gusb*, *Hmsb*, *Hprt*, *Impdh2, Mapk1*, *Oaz1*, *Pgk1*, *Ppia*, *Rpl13a*, *Rplp0*, *Sdha*, *Tbp*, *Tfrc*, and *Ywhaz*.

In our study, we utilized data derived from bulk RNA sequencing or microarray expression analysis (apart from one single cell RNA sequencing data set for ganglion cells). Single cell RNA sequencing has become an invaluable technique in gene expression profiling; in the retina, for example, single cell RNA sequencing has been proven extremely beneficial for identifying and clustering retinal cell types or even pinpointing molecular signatures of disease states (Macosko et al., 2015; Shekhar et al., 2016; Daniszewski et al., 2018; Laboissonniere et al., 2019; Lukowski et al., 2019; Menon et al., 2019). In this study, we were interested in measuring expression of a large cohort of IRD genes. Whereas RNA sequencing and microarrays typically detect expression of thousands of genes, single cell RNA sequencing data sets sometimes only cover a few hundred genes. The early stage single cell data sets had particularly low coverage and were not included in this study.

### Estimation of Photoreceptor Component in Non-photoreceptor Transcriptomes

Rod photoreceptors comprise ∼80% of retinal cells (Jeon et al., 1998) and represent the major source of contamination in other retinal cell types. To estimate photoreceptor contamination in the transcriptome of non-photoreceptor samples, we used expression level of *Rho* (a highly expressed rod specific gene). The ratio of *Rho* expression in a given sample versus age matched photoreceptor samples was used to estimate the photoreceptor component of the transcriptome in the given sample. To obtain the pure sample component of the non-photoreceptor transcriptomes, the photoreceptor components were taken away.

### Immunohistochemistry and TUNEL Stain

Mice were sacrificed, eyes enucleated and fixed in 4% paraformaldehyde in PBS for 4 h at 4°C. Eyes were washed in PBS, cryoprotected in 10, 20, and 30% sucrose in PBS, embedded in OCT (VWR), cryosectioned (12 μm), thaw-mounted onto polysine slides (Thermo Fisher Scientific) and stored at −20°C. Serial sections were taken in the optic nerve area. Immunocytochemistry was performed as described before (Palfi et al., 2016). Sections were incubate with primary antibodies (Table 1) overnight at 4°C, then incubated with secondary antibodies conjugated with either FITC, Alexa-Fluor-488, Cy3, and Alexa-Fluor-647 (Jackson ImmunoResearch Laboratories) in 1:400 dilution for 2 h, at RT and nuclei counterstained with DAPI. TUNEL staining was completed using the TMR red in situ cell death detection kit (Roche), as per the manufacturer’s protocol. Subsequent to TUNEL staining, sections were processed for immunocytochemistry. Sections were covered using Hydromount (National Diagnostics).

**TABLE 1:** Antibodies used in this study.

Primary antibody target	Number	Species	Dilution	Source
Rhodopsin	4D2	Mouse	1:200	Robert Molday, University of British Columbia, Vancouver, BC, Canada
Arrestin 3	AB15282	Rabbit	1:200	Merck
CRALBP	Ab15051	Mouse	1:200	Abcam
PKCα	P4334	Rabbit	1:2000	Sigma
CHX10	Sc-374151	Mouse	1:200	Santa Cruz
PAX6	PRB-278P	Rabbit	1:200	Biolegend
MAP2	Ab592	Chicken	1:600	Abcam
Calbindin	A85359	Chicken	1:200	Antibodies.com Ltd.
CTBP2	612044	Mouse	1:200	BD Biosciences
RBPMS	ABN1376	Guinea Pig	1:400	Millipore
Tulp1	M-tulp1N antibody	Rabbit	1:200	Stephanie A. Hagstrom Cleveland Clinic, Cleveland, OH
GABA	A2052	Rabbit	1:500	Sigma
COX4	Ab16056	Rabbit	1:200	Abcam

### Microscopy and Analysis

Fluorescent microscopy was carried out utilizing an Olympus IX83 inverted motorized microscope (cellSens v1.9 software) equipped with a SpectraX LED light source (Lumencor) and an Orca-Flash4.0 LT PLUS/sCMOS camera (Hamamatsu). Multi-channel fluorescence images were acquired as separate 16 bit gray-value images with fluorescence colors assigned and channels superimposed. In a given observation method, the same settings/operations were applied to all images. Pan-retinal images were produced from 10× magnification (plan fluorite objective) images with lateral frames stitched together in cellSens. Single frame images were taken using 20× and 40× plan super apochromat objectives using enhanced focal imaging (EFI) with 5–8 Z-slices. Sections were taken from areas adjacent (within 200 μm) to the optic nerve and randomly allocated for various analyses.

Measurements were taken in images from the central half of the retinas using comparable areas and similar analysis windows; object numbers were normalized to length of retina. Typically, four sections/eye were analyzed, and four measurements per section were made. All measurements were made using cellSens software (Olympus). Thickness of retinal layers was determined in 10× magnification stitched images from sections stained with COX4 immunocytochemistry. PAX6-positive cells were counted in 10× magnification stitched images using automatic image analysis; 2D deconvolution and Count and Measure tool. Bipolar (CHX10 positive) cells were manually counted in single frame 40× images. TUNEL-positive cells were counted in 10× pan-retinal stitched images using manual counting. For intensity measurements, the area of interest was delineated using the Closed Polygon tool and mean fluorescence intensity, i.e., mean 16-bit gray value determined.

Representative images for figures were exported from cellSens as individual fluorescence channels and post-processed in Photoshop CS6 (Adobe). In a given observation method, the same settings/operations were applied to all images both in cellSens and Photoshop. The only exception from this was in images co-labeled with TUNEL stain and immunocytochemistry, where the aim was to highlight immune-positive cells (independent to their original signal intensity) in order to facilitate localization of TUNEL-positive cells.

### TULP1 Interactors

Potential TULP1 interactor genes were predicted with PrePPI (Zhang et al., 2012) and STRING (Szklarczyk et al., 2019) using default settings for PrePPI and medium confidence for STRING and resulted in 182 human (179 mouse) and 68 interactors, respectively (Supplementary Table 1); 22 interactors were predicted by both resources (Supplementary Table 1). Eleven validated interactors (ACTB, ACTG1, AXL, CTBP2, DNM1, FYN, GRB2, MAP1B, MERTK, NCK1, and TYRO3) were taken from the literature; seven of them were also predicted with PrePPI or SRING (Supplementary Table 1). Combined lists of 229 interactors (predicted by at least one resource or validated) and 33 interactors (predicted by both resources or validated) were assembled (Supplementary Table 1). Utilizing the GEO open source retinal expression data sets, TULP1 interactors were ranked by normalized expression level for selected retinal cell types and postnatal ages, and the top 20% highest expressed interactors (46) from each cell type selected for analysis. The high confidence list of 33 interactors were also analyzed separately. Heat maps for selected data sets were generated with the pheatmap package in *R*^2^. Default settings were applied except for scaling in row direction and Pearson correlation as the distance matrix. Venn diagrams were constructed using a Venn diagram tool^3^.

### Statistical Analysis

Datasets were analyzed with Shapiro–Wilk test for normality before applying parametric tests. Paired sample *t*-test or a one-way analysis of variance (ANOVA) with a Tukey’s multiple comparison *post hoc* test was used to compare study groups; *n* numbers varied between 3 and 6. Brown–Forsythe test was utilized to determine the robustness of testing equality of means. The homogenous subset test was also included in the measurements in order to establish the likelihood of encountering type I error. A *p*-value of less than 0.05 was considered statistically significant. Statistical analyses were carried out using IBM SPSS and Minitab software; Microsoft Excel was used to plot the charts.

## Results

### TULP1 Expression in the Mouse Retina

**FIGURE 1:**
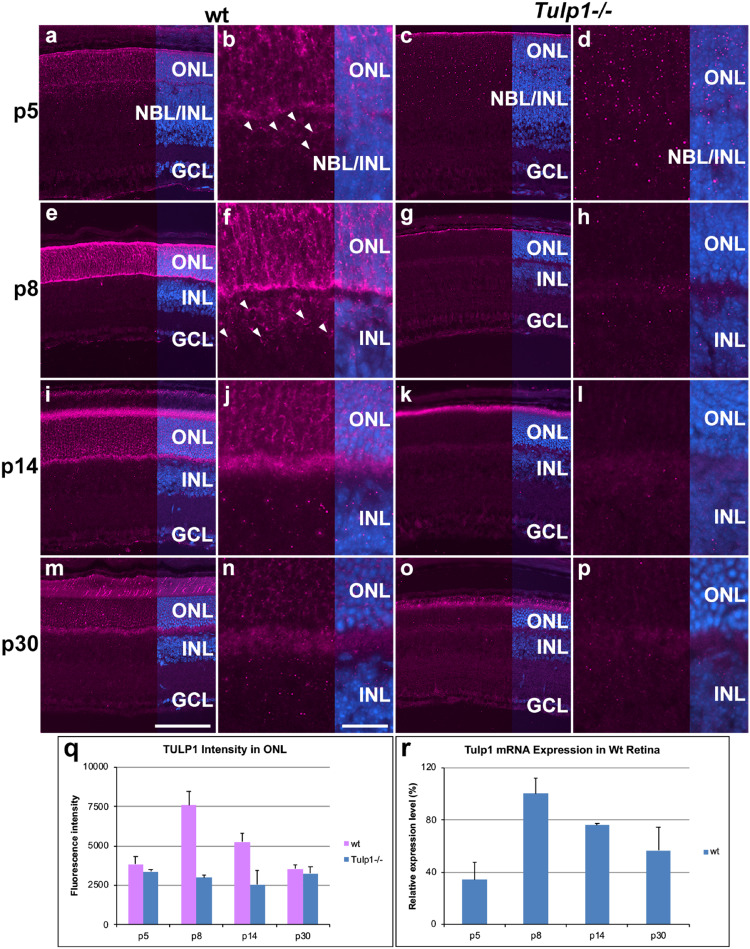
TULP1 expression during postnatal development of the retina. Retinas from wt and *Tulp1-/-* mice were taken at p5, p8, p14, and p30 (*n* = 4). Sections were labeled with TULP1 (purple) immunocytochemistry and counterstained with DAPI (blue). **(a,c,e,g,i,k,m,o)** low magnification overview; **(b,d,f,h,j,l,n,p)** high magnification view of the ONL/INL boundary. Arrowheads indicate TULP1-positive cells in the NBL/INL **(b)** and INL **(f)**. TULP1 label intensities were quantified in the outer nuclear layer (ONL; **q**) from microscope images using cellSens software and results are given in a bar chart; bars represent mean + SD (*n* = 4). **(r)** *Tulp1* RTqPCR was carried out using *Actb* control for normalization; data are provided in a bar chart; bars represent mean + SD (*n* = 3). INL, inner nuclear layer, NBL, neuroblast layer; GCL, ganglion cell layer. Scale bar **(m)**: 100 μm. Scale bar **(n)**: 20 μm.

Expression of *Tulp1* has thus far solely been associated with photoreceptors in the mouse retina (Hagstrom et al., 1999; Ikeda et al., 2000). Utilizing TULP1 immunohistochemistry (M-tulp1N antibody) (Hagstrom et al., 1999) in wt mouse retinas, TULP1 expression was observed in both photoreceptor and non-photoreceptor cells. We found evidence of photoreceptor expression by p5, an earlier timepoint than previously analyzed (Figures 1a,b). TULP1 was distributed in the perikaryon, inner segment and synapse of developing photoreceptors by p8 (Figures 1e,f), which became largely confined to the inner segments and the synaptic ribbons in wt retinas by p14–p30 (Figures 1i,j,m,n); reflecting previous studies (Hagstrom et al., 1999; Xi et al., 2005). TULP1 expression in the ONL peaked at p8 (*p* < 0.001) (Figure 1q). Notably, weak but consistent TULP1 expression was observed in the neuroblast and inner nuclear layers (NBL/INL) at p5 (Figure 1b) and in the INL at p8 (Figure 1f) but not at p14 (Figure 1j) or p30 (Figure 1n). No TULP1 expression was found in *Tulp1-/-* retinas as anticipated (Figure 1), although background label was observed in the tips of degenerating outer segments at p14 (Figure 1k) and p30 (Figure 1o) as also observed in outer segment tips of wt retinas. RT-qPCR analysis from wt retinal RNA (Figure 1r) mirrored the TULP1 expression profile in the ONL (Figure 1q) with peak expression at p8.

**FIGURE 2:**
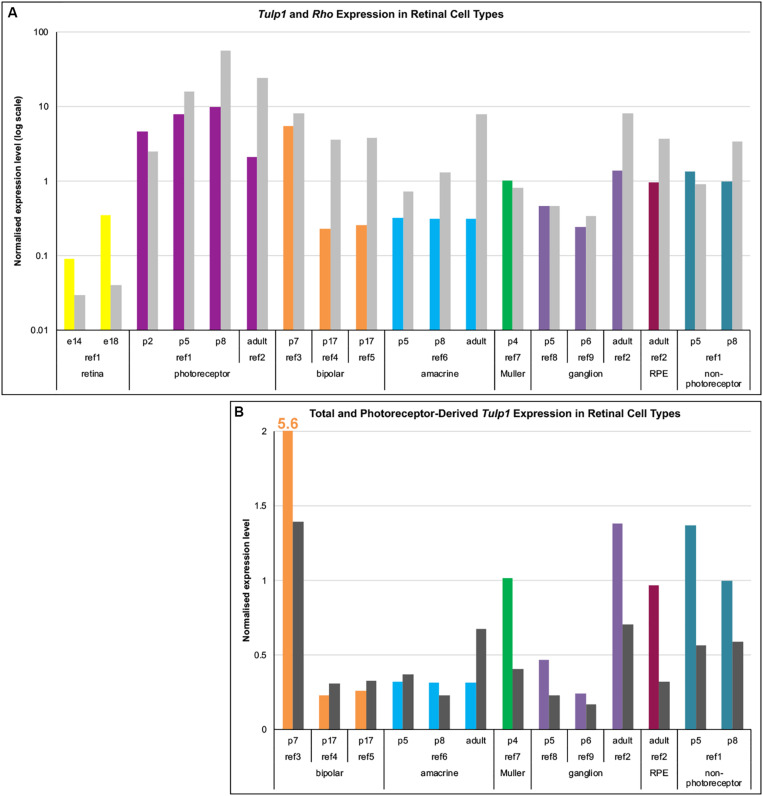
Expression analysis of *Tulp1* in retinal cell types. Raw expression data were obtained from GEO (NCBI) using GSE71462 (ref1), GSE19304 (ref2), GSE127771 (ref3), GSE80232 (ref4), GSE81903 (ref5), GSE33088 (ref6), GSE86199 (ref7), GSE115404 (ref8), GSE90652 (ref9) data sets. Expression levels were normalized using the average expression of 17 housekeeping genes. **(A)** Normalized expression values of *Tulp1* (color coded for different cell types) and *Rho* (gray) from various retinal cell type samples are given in a bar chart. **(B)** The total (color coded for different cell types) and the photoreceptor component (dark gray; estimated using *Rho* expression) of *Tulp1* expression in various samples is given in a bar chart.

As TULP1 levels in the inner retina were low, to probe the identity of positive cells we explored *Tulp1* mRNA expression. Data mining of public mRNA expression data sets (GSE71462, GSE19304, GSE127771, GSE80232, GSE81903, GSE33088, GSE86199, GSE115404, GSE90652) from purified populations of specific retinal cell types was undertaken employing the NCBI GEO resource. Expression levels were normalized using the average expression of 17 housekeeping genes. Normalized expression of *Tulp1* (and *Rho*, a rod specific gene) in various retinal cell types is given in Figure 2A. In photoreceptors (Figure 2A), the *Tulp1* expression profile was similar to that found with RTqPCR from whole retinas (Figure 1r) and TULP1 immunocytochemistry in the ONL (Figure 1q) with peak of expression being observed at p8. Non-photoreceptor retinal cell types also expressed *Tulp1*, although typically at lower levels compared to photoreceptors (Figure 2A).

As the majority of retinal cells are rod photoreceptors (from ∼p5 onward) (Jeon et al., 1998), they represent the major source of contamination when separating non-photoreceptor cells. To estimate the photoreceptor component of *Tulp1* expression in non-photoreceptor samples, we utilized expression level of *Rho* (Figure 2A). Estimated photoreceptor contamination ranged between ∼2–30% (11.9 ± 10.6%) in the non-photoreceptor samples and accounted for various amounts of *Tulp1* expression (Figure 2B). Results from this analysis suggested *bona fide Tulp1* expression in p7 bipolar, p4 Muller, developing and adult ganglion and adult RPE cells (Figure 2B). p5–p8 non-photoreceptors (the cells remaining after sorting photoreceptors) were also positive for *Tulp1* expression supporting the cell-specific data (Figure 2B). In other cell types, e.g., amacrine cells, it appeared that all *Tulp1* expression was accounted for by the photoreceptor component suggesting no actual *Tulp1* expression in these cells (Figure 2B). *Tulp1* was also expressed in the embryonic retina (Figure 2A) but the identity of the positive cell types was unknown.

### Development, Degeneration and Remodeling of the *Tulp1-/-* Retina

Our results revealed that TULP1 was present not only in photoreceptors (p5–p30) but in the NBL/INL at p5 and p8 in the mouse retina. Additionally, bioinformatic analysis also suggested *Tulp1* expression in various inner retinal cell types in wt mice. Therefore, we hypothesized, that the *Tulp1-/-* retina could be compromised by primary defects not only in the outer retina as previously described (Hagstrom et al., 1999; Ikeda et al., 2000) but in the inner retina too. Development, degeneration and remodeling in such a retina may differ from retinas affected solely by a primary photoreceptor degeneration, that is driven by a gene whose expression is limited to photoreceptors. To test this hypothesis, we selected two gene knock-out mouse models; *Rho-/-* (Humphries et al., 1997) and *Rds-/-* (Sanyal et al., 1980) as disease controls, where the causative genes are considered to be specific to photoreceptor cells. We compared the architecture of retinas from *Tulp1-/-*, disease control (*Rho-/-* and *Rds-/-*) and wt mice using a range of cellular markers. Mice were on a C57/BL6J background, except for *Rds-/-*, which was on a O20/A background.

### Gross Retinal Architecture and Outer Retina

Detailed findings of the gross retinal architecture and the outer retina are given in Supplementary Results including Supplementary Figures 1–5. The overall architecture of IRD (*Tulp1-/-*, *Rho-/-*, and *Rds-/-*) retinas was similar and involved a significant loss of the ONL from p14 (Supplementary Figure 1). Regarding the outer retina, significant shortening of photoreceptor segments by p14 (Supplementary Figure 2), mislocalization of RHO (Supplementary Figure 2), delayed cone development (Supplementary Figure 3), thinning of the C-terminal binding protein 2 (CTBP2)-positive synaptic region in the outer plexiform layer (OPL) by p14 (Supplementary Figure 4) and ectopic sprouting of horizontal cell neurites at p8–p14 (Supplementary Figure 5) were observed. While some significant differences between IRD retinas were detected, for example, different CTBP2 expression profiles in the OPL (Supplementary Figure 4); most alterations in photoreceptors (Supplementary Figures 2, 3), horizontal cells (Supplementary Figure 5) and the OPL (Supplementary Figure 4) were similar between IRD retinas.

### Inner Retina

**FIGURE 3:**
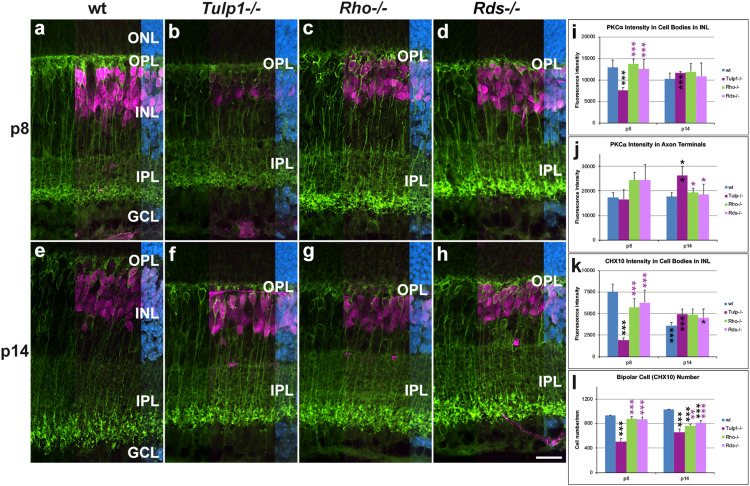
Bipolar cells detected with PKCα and CHX10 immunocytochemistries. **(a–h)** Retinal sections from wt, *Tulp1-/-*, *Rho-/-*, and *Rds-/-* mice at p8 and p14 (*n* = 6) were co-labeled with PKCα (green) and CHX10 (purple, overlaid on the right) and counterstained with DAPI (blue; overlaid on the far right). CHX10 labels all bipolar cell bodies while PKCα labels rod bipolar cells only. Fluorescence label intensities and cell numbers were determined in microscope images using cellSens software and results are given in bar charts; bars represent mean + SD. **(i,j)** PKCα intensity in the cell bodies and in axon terminals in inner plexiform layer (IPL), respectively (*n* = 4). **(k)** CHX10 intensity in cell bodies (*n* = 4). **(l)** Quantification of CHX10-positive cells (*n* = 6). ONL: outer nuclear layer, OPL, outer plexiform layer; INL, inner nuclear layer; GCL ganglion cell layer. Scale bar **(h)**: 20 mm. *p < 0.05; **p < 0.01; ***p < 0.001 (ANOVA). Black stars above bars refer to differences between IRD and wt mice, purple stars above bars refer to differences between *Tulp1-/-* and the other IRD mice; black stars within bars refer to a difference compared to the previous time point.

Given the interesting observation of *Tulp1* expression in the murine inner retina, a more detailed analysis was undertaken in bipolar, Muller, ganglion, and amacrine cells. Protein kinase Cα (PKCα) (Figure 3) labels rod bipolar cells and ceh-10 homeodomain containing homolog (CHX10) labels the cell bodies of all bipolar cells (Figure 3). PKCα intensities were measured in cell bodies (INL, Figure 3i) and axon terminals (IPL, Figure 3j). PKCα expression decreased in *Rho-/-*, *Rds-/-*, and wt retinas, while it increased in *Tulp1-/-* retinas between p8 and p14 (Figures 3i,j). Additionally, a marked loss of PKCα-positive dendritic arborization from p8 (Figures 3a–h) and compromised axon terminals at p14 (Figures 3e–h) were evident in all three IRD models. CHX10 labeling was similar in intensity between wt, *Rho-/-*, and *Rds-/-* (Figures 3a,c,d,k) retinas peaking at p8. In contrast, CHX10 expression was significantly less in *Tulp1-/-* (Figures 3b,k) compared to wt, *Rho-/-*, and *Rds-/-* retinas at p8. CHX10 intensities increased in *Tulp1-/-* (Figure 3k), while decreasing in the other retinas between p8 and p14 (Figure 3k). There were significantly fewer CHX10-positive cell bodies in *Tulp1-/-* (Figures 3a–d,l) compared to all other retinas at p8 and in the IRD retinas compared to wt p14 (Figure 3l). In summary, the above measures of bipolar cells were similar between wt, *Rho-/-*, and *Rds-/-* and significantly different from *Tulp1-/-* retinas.

**FIGURE 4:**
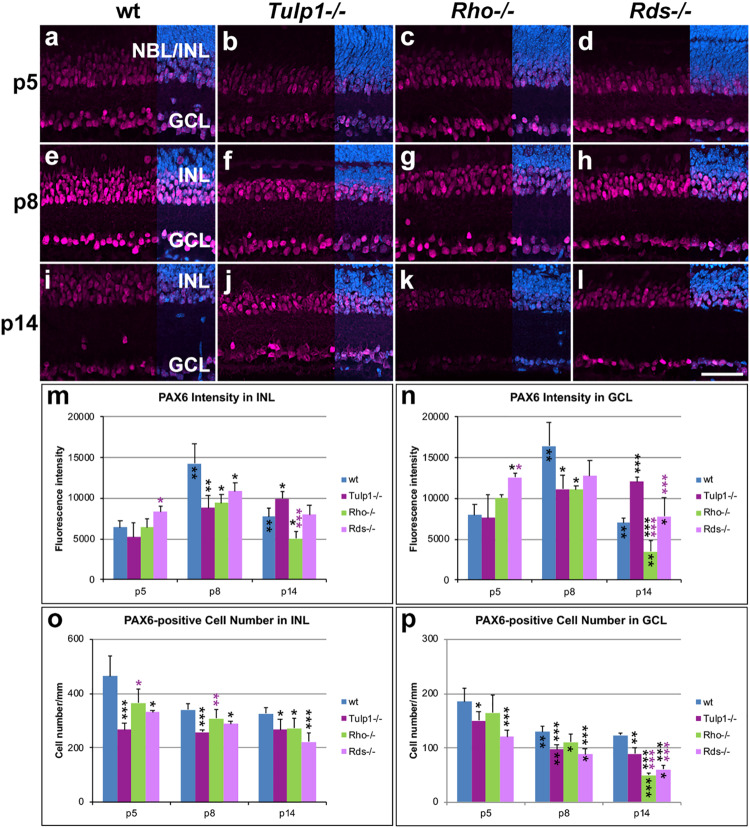
Amacrine and ganglion cells stained with PAX6 immunocytochemistry. Retinas from wt, Tulp1-/-, Rho-/-, and Rds-/- mice were taken at p5, p8, and p14 (n = 5–6). **(a–l)** Sections were labeled with PAX6 immunocytochemistry (purple) and counterstained with DAPI (blue; overlaid on right). PAX6 label intensities (**m,n**; *n* = 4) and PAX6-positive cell numbers (**o,p**; *n* = 5) were quantified in the inner nuclear layer (INL) and ganglion cell layer (GCL) from microscope images using cellSens software and results are given in bar charts; bars represent mean + SD. NBL, neuroblast layer. Scale bar **(l)**: 50 mm. *p < 0.05; **p < 0.01; ***p < 0.001 (ANOVA). Black stars above bars refer to differences between IRD and wt mice, purple stars above bars refer to differences between *Tulp1-/-* and the other IRD mice; black stars within bars refer to a difference compared to the previous time point.

Paired Box 6 (PAX6) immunocytochemistry labels amacrine and ganglion cells in the developing mouse retina. In the INL and ganglion cell layer (GCL) of wt, Rho-/-, and Rds-/- retinas, expression of PAX6 was low at p5, reached its highest intensity at p8 and decreased by p14 (Figure 4). In *Tulp1-/-* retinas, the expression profile differed substantially; expression increased from p5 to p14 and peak expression was delayed from p8 to p14. PAX6-positive cell numbers decreased in all retinas from p5 to p14; IRD retinas had lower numbers compared to wt.

**FIGURE 5:**
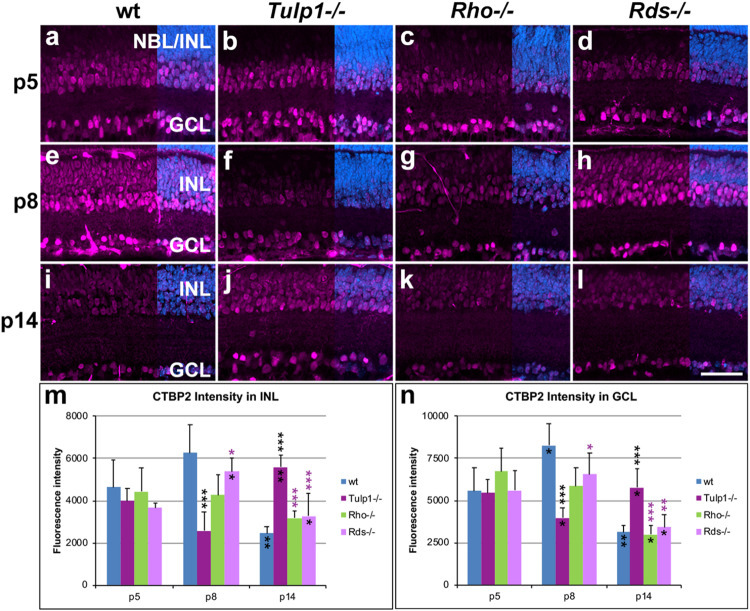
CTBP2 labeling in INL and GCL. Retinas from wt, *Tulp1-/-*, *Rho-/-*, and *Rds-/-* mice were taken at p5, p8, and p14 (*n* = 5–6). **(a–l)** Sections were labeled with CTBP2 immunocytochemistry (purple) and counterstained with DAPI (blue; overlaid on the right). CTBP2 label intensities (*n* = 4) were quantified in the inner nuclear layer (INL; **m**) and ganglion cell layer (GCL; **n**) from microscope images using cellSens software, and results are given in bar charts; bars represent mean + SD. NBL, neuroblast layer, ONL, outer nuclear layer. Scale bar **(l)**: 50 mm. *p < 0.05; **p < 0.01; ***p < 0.001 (ANOVA). Black stars above bars refer to differences between IRD and wt mice, purple stars above bars refer to differences between *Tulp1-/-* and the other IRD mice; black stars within bars refer to a difference compared to the previous time point.

**FIGURE 6:**
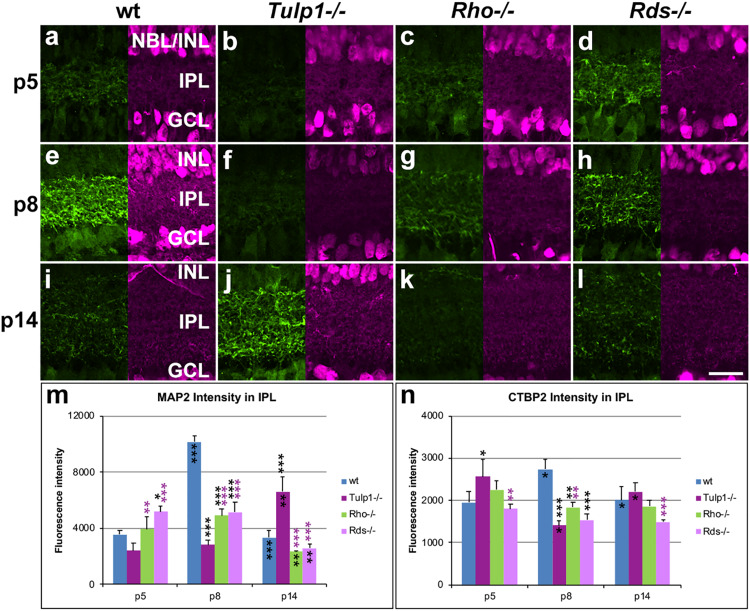
Inner plexiform layer (IPL) and ganglion cells. Retinas from wt, *Tulp1-/-*, *Rho-/-*, and *Rds-/-* mice were taken at p5, p8, and p14 (*n* = 5–6). **(a–l)** Sections were co-labeled with MAP2 (left; green) and CTBP2 (RYBEYE; right; purple) immunocytochemistries. MAP2 (*n* = 3; **m**) and CTBP2 (*n* = 3; **n**) label intensities in the inner plexiform layer (IPL) were quantified from microscope images using cellSens software and results are given in bar charts; bars represent mean + SD. INL, inner nuclear layer; NBL, neuroblast layer. Scale bar **(l)**: 20 mm. *p < 0.05; **p < 0.01; ***p < 0.001 (ANOVA). Black stars above bars refer to differences between IRD and wt mice, purple stars above bars refer to differences between *Tulp1-/-* and the other IRD mice; black stars within bars refer to a difference compared to the previous time point.

The CTBP2 gene encodes two alternative transcripts producing two distinct proteins; CTBP2 (C-terminal binding protein 2, a transcriptional repressor) and RIBEYE (a major component of the synaptic ribbons in the retina). The CTBP2 antibody used detects both CTBP2 and RIBEYE. CTBP2 is located in nuclei, while RIBEYE in synaptic regions (OPL and inner plexiform layer /IPL/), hence the two proteins are spatially separated and therefore readily distinguished. CTBP2 labels cell bodies predominantly in the INL and GCL (Figure 5). Expression in cell bodies peaked at p8 in wt and *Rds-/-* INLs (Figure 5m) and GCLs (Figure 5n); in contrast CTBP2 labeling of cell bodies was at its lowest level in *Tulp1-/-* retinas at p8 and peaked at p14 (Figures 5m,n). CTBP2 expression intensity levels in cell bodies decreased in *Rho-/-* retinas from p5 to p14 (Figures 5m,n). In the IPL, CTBP2 (RIBEYE) is localized to the synaptic ribbons of bipolar cells. CTBP2 labeling in the IPL peaked in wt retinas at p8 (Figures 6e,n), while it was lowest in *Tulp1-/-* retinas at this time point (Figures 6f,n). CTBP2 label intensities in IPL were similar at all ages in *Rho-/-* and *Rds-/-* retinas (Figure 6n).

**FIGURE 7:**
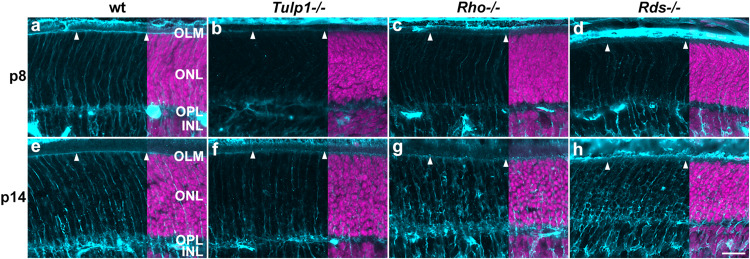
Muller cell labeling (CRALBP) in the outer retina. Retinas from wt, *Tulp1-/-*, *Rho-/-*, and *Rds-/-* mice were taken at p8 and p14 (*n* = 5–6). **(a–h)** Sections were labeled with CRALBP immunocytochemistry (light blue) and counterstained with DAPI (purple; overlaid on the right). ONL, outer nuclear layer; OPL, outer plexiform layer; INL, inner nuclear layer. Arrowheads point to the OLM. Scale bar **(h)**: 20 m.

Muller cells, the primary macroglia in retina were labeled using cellular retinaldehyde-binding protein (CRALBP). CRALBP expression was low in p5 retinas and increased by p8 in all but *Tulp1-/-* (Supplementary Figure 6). In wt retinas, Muller processes labeled strongly and spanned the retina radially from the outer limiting membrane (OLM) to the inner limiting membrane (ILM) by p8 (Supplementary Figure 6e). In the ONL, the radial processes reached the OLM and formed a CRALBP-positive continuous layer at p8 (Supplementary Figure 6e and Figure 7a); this pattern was similar at p14 (Supplementary Figure 6i and Figure 7e). In *Tulp1-/-* retinas, CRALBP labeling was weak in the ONL-IPL region at p8 (Supplementary Figure 6f and Figure 7b), the radial processes in the ONL did not reach the OLM until p14 (Figure 7f). The CRALBP-positive layer in the OLM was weak at p8, and less intense at p14 compared to wt (Figures 7b,f). In *Rho-/-* and *Rds-/-* retinas, CRALBP expression in the INL-IPL region was between wt and *Tulp1-/-* at p8 (Figures 7c,d). Radial Muller glia processes in the ONL were straight and ran between the columns of photoreceptor nuclei in wt retinas (Figures 7a,e); in contrast, they appeared to enclose some photoreceptor cell bodies in IRD retinas (Figures 7b–d,f–h). The severity of this phenotype increased from *Tulp1-/-* to *Rho-/-* and to *Rds-/-* and from p8 to p14 (Figure 7). These latter findings are in line with (Sakami et al., 2019) suggesting that Muller cells were the primary effectors of photoreceptor phagocytosis in P23H-RHO mice (analyzed at p14). The current study suggests that this event has been initiated in *Tulp1-/-*, *Rho-/-*, and *Rds-/-* retinas by p8. Other studies also reported remodeling of Muller cells but at later time points; for example, glutamine synthetase expression decreased by p20 and GFAP increased from p15 in Muller cells of the rd10 retina (Roche et al., 2016). GFAP increased from p18 to 30 in the rd1 retina (Strettoi et al., 2003).

Ganglion cells were labeled for microtubule associated protein 2 (MAP2) and RNA binding protein mRNA processing factor (RBPMS). MAP2 labeled the dendritic tree of ganglion cells in the IPL (Figure 6), while RBPMS predominantly localized to the cell bodies and main dendritic branches of ganglion cells (Supplementary Figure 7). Expression of MAP2 (Figure 6m) and RBPMS (Supplementary Figure 7m) was similar in wt retinas; expression was low at p5, peaked at p8 and decreased by p14. MAP2 expression was significantly less in IRD compared to wt retinas at p8 (Figure 6m). Notably, MAP2 labeling was significantly less at p8 and higher at p14 in *Tulp1-/-* compared to *Rho-/-* and *Rds-/-* retinas (Figure 6m). In contrast, less differences were found in RBPMS expression levels between IRD and wt retinas (Supplementary Figure 7m), however, the number of RBPMS-positive cells was higher in *Tulp1-/-* retinas at p5 and p8. In summary, the observations suggest that development of ganglion cells, in particular, ganglion cell dendrites in the IPL are altered in IRD retinas, and differ significantly between *Tulp1-/-*, and *Rho-/-* and *Rds-/-* retinas.

In summary, in the inner retina, expression of PKCα, CHX10, CTBP2 (and RYBEYE) and MAP2, increased in *Tulp1-/-* retinas from p8 to p14, while their expression was maintained or decreased in *Rho-/-*, *Rds-/-*, and wt retinas during the same period suggesting an altered development of *Tulp1-/-* retinas compared to the other retinas analyzed.

### TUNEL-Positive Cells

**FIGURE 8:**
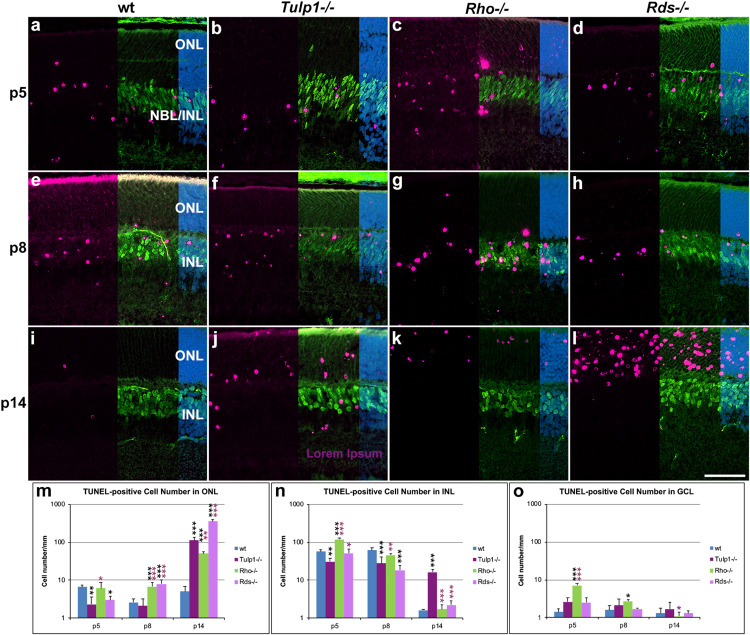
Combined TUNEL stain and CHX10 immunocytochemistry in the ONL – INL region. Retinas from wt, *Tulp1-/-*, *Rho-/-*, and *Rds-/-* mice were taken at p5, p8, and p14 (*n* = 3). **(a–l)** Sections were co-labeled with TUNEL stain (purple) and CHX10 (green) immunocytochemistry (overlaid on the right side), and counterstained with DAPI (blue; overlaid on the far right). Quantification of TUNEL-positive cells in the outer nuclear layer (ONL; **m**), inner nuclear layer (INL; **n**) and ganglion cell layer (GCL; **o**) were carried out from microscope images using cellSens software and results are given in bar charts; bars represent mean + SD. NBL, neuroblast layer. Scale bar **(l)**: 50 mm. *p < 0.05; **p < 0.01; ***p < 0.001 (ANOVA). Black stars refer to differences between IRD and wt mice, purple stars refer to differences between *Tulp1-/-* and the other IRD mice.

TUNEL (terminal deoxynucleotidyl transferase dUTP nick end labeling) staining was performed to enable visualization of apoptotic cells. Cells were co-labeled for CHX10 (Figure 8) and PAX6 (Supplementary Figure 8) to aid localization of TUNEL-positive cells in the INL. At p5–p8, the majority of TUNEL-positive cells were in the NBL and bipolar cell region of the INL in both wt and IRD retinas (Figures 8a–h and Supplementary Figures 8a–h). The photoreceptor and GCLs also had TUNEL-positive cells but in lower numbers. The number of TUNEL-positive cells increased in the ONL of *Rho-/-* and *Rds-/-* retinas from p8 and in *Tulp1-/-* retinas from p14 (Figure 8m); this was mirrored by significantly decreased ONL thickness in the IRD retinas from p14 (Supplementary Figure 1q). Notably, significantly more TUNEL positive cells were found in the INL of *Tulp1-/-* compared to wt, *Rho-/-*, and *Rds-/-* retinas by p14 (Figure 8n), however, this was not reflected in decreased *Tulp1-/-* INL thickness at either p14 or p30 (Supplementary Figure 1r).

The results indicate a large-scale photoreceptor apoptosis in the three IRD models from p8–p14, timeframes that are somewhat earlier than detected previously in *Rds-/-* retinas from p14 (Portera-Cailliau et al., 1994) and in *Tulp1-/-* retinas from p18 (Ikeda et al., 2000). Interestingly, by p14, apoptosis is largely over in the INL of wt, *Rho-/-*, and *Rds-/-*, but not in *Tulp1-/-* retinas. As the INL thickness did not decrease significantly in *Tulp1-/-* compared to the other retinas by p30, the presence of significant apoptosis at p14 may probably be a sign of a delayed developmental process rather than progressive degeneration of the INL in *Tulp1-/-* retinas at this stage. Lower number of apoptotic cells in *Tulp1-/-* compared to wt INLs at p5-p8 are also suggestive of delayed developmental apoptosis in *Tulp1-/-* retinas.

### Cell Type Specific TULP1 Interactomes in the Retina

Our analysis indicated that *Tulp1* was expressed, apart from photoreceptors, in, bipolar, Muller, ganglion, and RPE cells. Additionally, early changes in the *Tulp1-/-* retina exhibited specific, *Tulp1-*linked perturbations in bipolar, Muller, amacrine and ganglion cells. As the function(s) of TULP1 are not clearly established, we aimed to interrogate the potential roles that TULP1 might have in these retinal cell types.

**FIGURE 9:**
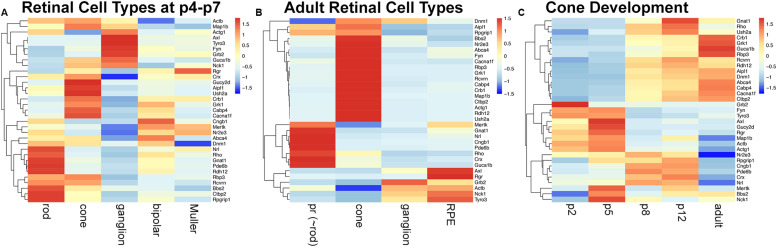
mRNA expression of a high-confidence TULP1 predicted interactome in various retinal cell types. A list of 33 TULP1 interactors were assembled from predicted (by both PrePPI and STRING) and 11 validated interactors. Expression of their corresponding genes in various retinal cell types was determined from Gene Expression Omnibus data sets; GSE97534, GSE59201, GSE115404, GSE127771, GSE86199, GSE19304, and is given in row-scaled clustered heatmap representation in some p4-p7 **(A)** and adult **(B)** retinal cell types and in developing cone cells at p2, p5, p8, p12, and adult **(C)**. Note that in panel **(A)**, rod, cone, and ganglion cells are from p5, bipolar cells are from p7 and Muller cells are from p4 retinas. pr: photoreceptor. A zoom-enabled version of this figure is given as Supplementary Figure 9.

Tubby like protein 1 (TULP1) interactors were predicted with PrePPI (Zhang et al., 2012) and STRING (Szklarczyk et al., 2019) and resulted in 179 and 68 interactors, respectively (Supplementary Table 1); 22 interactors were predicted by both resources (Supplementary Table 1). Eleven validated interactors were taken from the literature (Supplementary Table 1); seven of which were also predicted with PrePPI or SRING. Proteins were merged into a medium confidence list of 229 TULP1 predicted interactors (Supplementary Table 1). A high-confidence list of 33 TULP1 interactors from the above list includes interactors validated or predicted by both PrePPI and STRING. Cell type-specific mRNA expression levels of TULP1 predicted interactor genes were determined in retinal cell types where bioinformatic analysis suggested expression of *Tulp1*; GEO expression data sets (GSE97534, GSE59201, GSE115404, GSE127771, GSE86199, GSE19304) were used. Expression levels of high-confidence predicted interactor genes are given in heatmaps for p4–p7 and adult cell types (Figures 9A,B), as well as, during postnatal development of cones (Figure 9C). A zoom-enabled pdf version of this figure is given in Supplementary Figure 9). TULP1 predicted interactor genes from the medium-confidence list were ranked by expression level in retinal cell types and expression of the highest expressed 20% predicted interactor genes from each cell type further analyzed by heat maps and Venn diagrams (Supplementary Figure 10).

Significant differences in expression of TULP1 predicted interactor genes between rod, cone, ganglion, bipolar, and Muller cells at p4–p7 (Figure 9A and Supplementary Figures 10a–c), and between photoreceptor (∼rod), cone, ganglion, and RPE cells from adult retinas (Figure 9B and Supplementary Figures 10d–f) were determined. During development of cone photoreceptors, few (∼10%) age-specific predicted interactors were found (Supplementary Figures 10g–i), although the expression pattern of the predicted interactor genes varied with age (Figure 9C and Supplementary Figure 10g).

Regarding expression of the high-confidence TULP1 interactor genes (Figure 9) in rods and cones, various functional groups were predicted. Cytoskeletal (including intracellular trafficking) interactor genes included *Actb*, *Actg1*, *Map1b*, *Rpgrip1*, *Dnm1*, *Bbs2*, and *Ush2a*. Ctbp2 and *Cabp4* are proteins linked to synaptic function. Outer segment interactors were found in rods and cones; e.g., *Rho*, *Gnat1*, *Pde6b*, and *Cngb1* in rods, and *Guca1b* and *Gucy2d* in cones. As the highest concentration of TULP1 is found in photoreceptor inner segments, TULP1 may be implicated in transport of outer segment proteins, similar to rod and cone opsins (Grossman et al., 2011). Alternatively, low levels of TULP1 may be present in the outer segments and could interact with predicted interactors *in situ*. Other photoreceptor- or retina-specific TULP1 interactor genes expressed in photoreceptors included transcription factors (*Nrl*, *Crx*, *Nr2e3*) and proteins such as *Rbp3*, *Abca4* (retinoid cycle), *Crb1* and *Aipl1*, often exhibiting differential expression between rod and cone photoreceptors (Figure 9).

Of note, expression of two functional groups of TULP1 interactor genes was predicted with high-confidence in retinal ganglion cells (Figure 9); some interactors were linked to signal transduction, for example, *Axl*, *Tyrp3*, *Grb2*, and *Nck1*, while others, that is, *Actb*, *Actg1*, *Dnm1*, and *Map1b* represented cytoskeletal interactors. TULP1 predicted interactor genes expressed in bipolar cells at p7, Muller cells at p4 and adult RPE cells (Figure 9) belonged to three functional groups; cytoskeleton (*Dnm1* in bipolar, *Actg1* in Muller and *Actb* and *Dnm1* in RPE cells), signal transduction (*Mertk* in bipolar and Muller, *Grb2* in Muller, and *Axl*, *Nck1*, *Tyro3*, and *Mertk* in RPE cells) and retinoid cycle (*Rgr* in RPE, Muller and bipolar, *Rbp3* in Muller and *Abca4* in bipolar and RPE cells).

### IRD Gene Expression in Early Postnatal Retinal Cell Types

**FIGURE 10:**
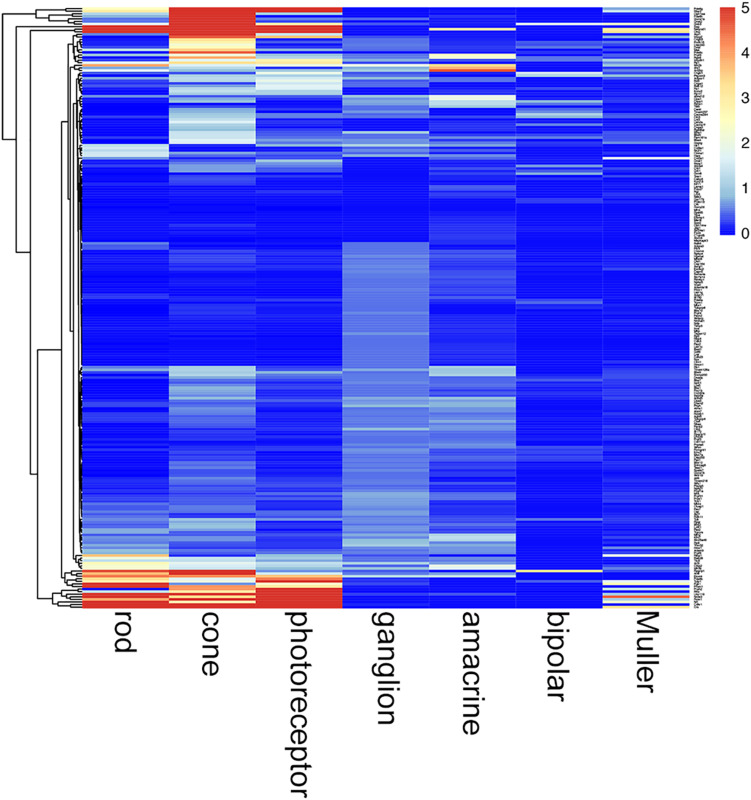
Cell type-specific expression of IRD genes in mouse retina at p4–p7. Retinal cell type-specific gene expression values of 235 mouse orthologs of a panel of 251 human IRD genes (Dockery et al., 2017) were determined using GSE59201, GSE97534, GSE71462, GSE33088, GSE127771, GSE86199, GSE115404 data sets from Gene Expression Omnibus. Photoreceptor contamination of non-photoreceptor samples was determined utilizing Rho expression and corresponding photoreceptor derived IRD gene expression values deducted from these samples. Expression values are given in a clustered heatmap format; pr, photoreceptor. A zoom-enabled version of this figure is given as Supplementary Figure 11.

*TULP1* is one of more than 300 genes causative of IRDs (RetNet - Retinal Information Network, 2019). In this study, we found that *Tulp1* was expressed in both photoreceptor and non-photoreceptor cells, in particular during development of the mouse retina. We queried how common this feature may be among other IRD genes. Using retinal expression data sets (GSE59201, GSE97534, GSE71462, GSE33088, GSE127771, GSE86199, GSE115404), normalized expression levels of 235 mouse orthologs of a panel of 251 human IRD genes (Dockery et al., 2017; Supplementary Table 2) were determined in selected retinal cell types at p4–p7 (Figure 10). The photoreceptor-derived component of the transcriptome was accounted for employing *Rho* expression in non-photoreceptor samples. The analysis suggests widespread expression of IRD genes in various, often multiple (for example, in photoreceptor, amacrine and ganglion cells), retinal cell types at p4–p7 (Figure 10; a zoom-enabled pdf version of this figure is given in Supplementary Figure 11). Of note, low level of *Rds* expression was detected in Muller cells; while only in these cells and at a low level, this is worth noting given we used *Rds-/-* retinas as disease controls.

## Discussion

*Tulp1* expression in photoreceptor and non-photoreceptor cells has been explored in the mouse retina and potential TULP1 interactors highlighted. While TULP1 expression has previously only been observed in photoreceptors in mice (Hagstrom et al., 1999; Ikeda et al., 2000; Xi et al., 2005), expression of human TULP1 has been detected in neuroblast, RPE, INL, and ganglion cells (Milam et al., 2000). In the current study, using immunocytochemistry we found evidence of TULP1 expression in the neuroblastic/INL region of the murine retina during the p5–p8 period (Figure 1), which has not been reported previously. Expression of TULP1 was also evident in the ONL at all ages analyzed (p5–p30) confirming prior studies (Hagstrom et al., 1999; Ikeda et al., 2000; Xi et al., 2005), but, moreover, demonstrating a developmental expression profile with *Tulp1* expression peaking at ∼p8, again not reported previously (Figure 1). While expression of TULP1 was found in occasional ganglion cells in humans (Milam et al., 2000), we did not detect protein expression in these cells in mice during the analyzed period. However, bioinformatic evaluation based on mRNA expression suggested that *Tulp1* was expressed in ganglion cells, as well as in RPE, bipolar and Muller cells in the mouse retina (Figure 2). Possibly, the affinity of the TULP1 antibody used in the study, i.e., M-tulp1N (Hagstrom et al., 1999), was not sufficient to detect the level of TULP1 in ganglion cells. Similarly, *Tulp1* expression was also detected in p5 ganglion cells using single cell transcriptome profiling (Rheaume et al., 2018). TULP1 has been implicated in MERTK-dependent phagocytosis in the RPE (Caberoy and Li, 2009), however, TULP1 in the RPE was suggested to originate from shedding of photoreceptor segments (Caberoy et al., 2010). Our bioinformatic analysis indicates that *Tulp1* is expressed in adult mouse RPE, in agreement with transcriptome analysis of human RPE/choroid (Whitmore et al., 2014). According to our study, *Tulp1* is expressed in p7 but not in p17 bipolar cells; no data were available for Muller cells. Notably, peak expression of *Tulp1* overlaps with differentiation, maturation and synaptic development of the mouse retina.

To explore the impact of TULP1 on the inner retina, we analyzed the architecture of the retina in *Tulp1-/-* mice in the p5–p14 window. This period coincides with a major part of postnatal development and peak expression of *Tulp1* in the mouse retina. As comparators we evaluated retinas from *Rho-/-* (Humphries et al., 1997) and *Rds-/-* (Sanyal et al., 1980) mice; *Rho* and *Rds* are believed to be expressed exclusively in photoreceptors in the retina. As such, in these mice, retinopathy is due to loss of photoreceptor-specific proteins. Therefore, any alterations in the inner retina should be the result of the primary photoreceptor degeneration. Notably, we detected an extremely low level of *Rds* expression in Muller cells (Figure 10). Most probably this represents photoreceptor contamination of the Muller cells analyzed (*Rho* expression was used to account for photoreceptor contamination). Alternatively, it could be caused by the close physical and functional contact (Sakami et al., 2019) between photoreceptors and Muller cells. When comparing *Tulp1-/-*, *Rho-/-*, and *Rds-/-* retinas, two main trends were identified.

Common alterations in all IRD retinas were observed including alterations in rod and cone photoreceptors, ectopic sprouting of horizontal cell neurites, distorted Muller glia labeling in the ONL, decreased immunoreactivity of CTBP2 in the OPL, compromised rod bipolar cell dendritic and axon terminal development, decreased bipolar cell numbers (Supplementary Figures 1–5) and a significant increase in TUNEL-positive photoreceptors by p8–p14. Similar features of early degeneration and remodeling have been described in other IRD mouse models (Strettoi and Pignatelli, 2000; Strettoi et al., 2002; Gargini et al., 2007; Grossman et al., 2009; Puthussery et al., 2009; Singh et al., 2014; Soto and Kerschensteiner, 2015; Roche et al., 2016).

Of note, we detected alterations in the architecture and development of the inner retina of *Tulp1-/-* mice, which differed from the specific, photoreceptor-driven degeneration observed in *Rho-/-* and *Rds-/-* retinas and were only characteristic of *Tulp1-/-* retinas. These included different expression profiles of CHX10 and PKCα in bipolar cells, of PAX6 and CTBP2 in INL and GCL and of MAP2 in IPL (Figures 3–7). Expression of PKCα, CHX10, CTBP2, and MAP2, increased in *Tulp1-/-* retinas from p8 to p14, while expression was maintained or decreased in *Rho-/-*, *Rds-/-*, and wt retinas during the same period. Additionally, there was a significant number of apoptotic cells in *Tulp1-/-* (but not in *Rho-/-*, *Rds-/-*, and wt) INLs at p14 (Figure 8).

Delayed peak expression (from p8 to p14) of the above cellular markers in *Tulp1-/-* retinas, may be a sign of altered development and maturation of bipolar, amacrine and ganglion cells. For example, MAP2 is a critical component for dendritic extension, branching and remodeling (Johnson and Jope, 1992) (Szebenyi et al., 2005) (Wang and Rubel, 2008). MAP2 upregulation is also a hallmark of activity-dependent stabilization of dendrites in cultured sympathetic neurons (Vaillant et al., 2002). Therefore, delayed MAP2 expression in the *Tulp1-/-* IPL may possibly indicate delayed development of synaptic connectivity in the *Tulp1-/-* retina. Delayed apoptosis in the INL also suggests that the normal developmental profile of the inner retina may be compromised in the *Tulp1-/-* retina. Overall, the data suggest that loss of *Tulp1* expression is causative of not only primary photoreceptor degeneration but possibly primary perturbation of inner retinal cells and circuitries.

As noted, the function of TULP1 has not been fully established; most studies have been focused on photoreceptor and RPE cells (see below). Trafficking between inner and outer segments (Hagstrom et al., 2001, 2012; Xi et al., 2005; Grossman et al., 2011), and cytoskeletal functions including vesicular transport in the synaptic region (Xi et al., 2007; Grossman et al., 2009, 2013; Wahl et al., 2016) are known functions in photoreceptor cells. These functions are in line with our analysis of cell type-specific expression of validated (such as *Actb*, *Actg1*, *Map1b*, and *Dnm1*) and predicted (such as *Bbs2*, *Rpgrip1*, and *Ush2a*) TULP1 interactor genes (Figure 9 and Supplementary Figure 10). Indeed, predicted cytoskeletal TULP1 interactor genes were expressed in all retinal cell types analyzed including bipolar, ganglion, Muller, and RPE cells. While TULP1’s role in the cytoskeleton has been described in the context of photoreceptors (see above), RPE (Caberoy et al., 2010) and DNM1 expression in the inner retina (Grossman et al., 2013), our study predicts TULP1 as a cytoskeletal interactor in all analyzed retinal cell types.

Notably, the data also highlight the retinoid visual cycle as a potential new TULP1-linked retinal function (Figure 9 and Supplementary Figure 10). Various proteins involved in aspects of the retinoid cycle (*Rbp3*, *Rgr*, and *Abca4*, and *Rpe65* and *Lrat* from the high- and medium-confidence predicted TULP1 interactor lists, respectively) were expressed in a number of retinal cell types, including photoreceptor (*Rbp3* and *Abca4*), bipolar (*Rgr* and *Abca4*), Muller (*Rgr* and *Rbp3*), and RPE cells (*Rgr*, *Abca4*, *Rpe65*, and *Lrat*). Expression of *Rgr* is typically linked to RPE and Muller cells, *Abca4* to photoreceptors and *Rbp3* to photoreceptor and retinoblastoma cells (Entrez). As such, our findings suggest some atypical sites of expression for these genes. However, other studies also detected uncommon expression for some of these genes; *Abca4* has been found expressed in RPE cells (Lenis et al., 2018) and *Rbp3* in bipolar and ganglion cells (Siegert et al., 2012). Additionally, as some of these cells are in close physical contact to each other in the retina, for example, photoreceptor and RPE, or photoreceptor and Muller cells, contamination between these cell types may happen and ultimately influence cell-specific gene expression data.

Signal transduction was another function newly predicted for TULP1 interactor genes in non-photoreceptor cells (Figure 9 and Supplementary Figure 10), for example, in RPE (*Axl*, *Nck1*, *Tyro3*, and *Mertk*), ganglion (*Axl*, *Tyro3*, *Grb2*, *Nck1*), bipolar (*Mertk*) and Muller (*Mertk* and *Grb2*) cells. Additionally, expression of a number of transcription factors that were predicted with high confidence as TULP1 interactors (*Nrl*, *Crx*, and *Nr2e3*) were detected (Figure 9). As these transcription factors were expressed in various retinal cell types, TULP1 may possibly be involved in regulation of development and maturation of the retina as indicated previously (Boggon et al., 1999; Milam et al., 2000; Grossman et al., 2009). In summary, cell type-specific expression of validated and predicted TULP1 interactor genes suggest differential utility of TULP1 in various cell types in mouse retina including bipolar and Muller cells, where expression of *Tulp1* was not recognized before. Some predicted TULP1-linked retinal functions, such as the retinoid visual cycle, or non-photoreceptor signal transduction have been highlighted for the first time. These studies were based on bioinformatic analysis of mRNA expression of predicted TULP1 interactor genes and so predicted TULP1 interactors will require validation by proteomic approaches. Nevertheless, our data highlight the potentially diverse roles that TULP1 may play in the retina.

The data suggest that *Tulp1* is expressed in various cell types in the retina; this may explain the severe phenotype of TULP1-linked IRD (Jacobson et al., 2014) and may be relevant from a therapeutic perspective. We interrogated how unique this feature might be among IRD genes (RetNet - Retinal Information Network, 2019). Indeed, expression of a significant proportion of ∼180 mouse IRD genes has previously been detected in adult retinal cell types (Siegert et al., 2012). However, to the best of our knowledge, cell type-specific expression of IRD genes has not been evaluated in the early postnatal mouse retina. As such, we analyzed the expression of 235 mouse orthologs of a panel of 251 human IRD genes (Dockery et al., 2017) in p4–p7 mouse retinal expression data sets and found widespread expression of IRD genes in various, often multiple retinal cell types at p4–p7 (Figure 10). Taking *Tulp1* as an example, this gene is primarily expressed in photoreceptor cells. However, it also appears to be expressed in a number of non-photoreceptor cell types as observed using both mRNA expression data and immunocytochemistry in this study. It is possible that restoring TULP1 levels solely in photoreceptors may not provide the level of therapeutic benefit expected. Data from Siegert et al. (2012), Jacobson et al. (2014) and the current analysis suggest more extensive expression in both the outer and inner retina for a significant number of IRD genes.

Current interest in ocular gene therapy is enormous with recent approval, by the FDA/EMA, of Luxturna, the first ocular gene therapy. Results from this study provide significant evidence of expression of *Tulp1* in murine retinal non-photoreceptor cells, in particular, during early postnatal development. These data correlate with human studies (Milam et al., 2000; Caberoy et al., 2010), additionally, they suggest significant roles for TULP1 in both photoreceptor and non-photoreceptor cells. A bioinformatic analysis of IRD genes suggests widespread expression of many other IRD genes in both inner and outer retinal cell types during early development. These findings raise the possibility that gene therapies targeting *TULP1* (Widgren et al., 2016; Chen et al., 2018) (Jacobson et al., 2014; Avela et al., 2019) and other similarly expressed IRD genes, may possibly need to restore expression of these genes in both photoreceptor and non-photoreceptor cells. Furthermore, early postnatal expression of these genes highlights a potential hurdle for future treatments of some IRDs.

## Data Availability Statement

All datasets presented in this study are included in the article/Supplementary Material.

## Ethics Statement

The animal study was reviewed and approved by the Animal Research Ethics Committee, University of Dublin, Trinity College.

## Author Contributions

AP: concept, experimental design, experiments, figures and artwork, writing the manuscript, and grant support. AY: experimental design, experiments, and writing the manuscript. PH: concept and grant support. KH: bioinformatics and writing the manuscript. GF: concept, experimental design, writing the manuscript, and grant support. All authors contributed to the article and approved the submitted version.

## Conflict of Interest

The authors declare that the research was conducted in the absence of any commercial or financial relationships that could be construed as a potential conflict of interest.

## Footnotes

1 https://github.com/khokamp/Perl-scripts

2 https://cran.r-project.org/web/packages/pheatmap/index.html

3 http://bioinformatics.psb.ugent.be/webtools/Venn/
