# Supplementary material for: Non-photoreceptor Expression of Tulp1 May Contribute to Extensive Retinal Degeneration in Tulp1-/- Mice
Source: Front Neurosci. 2020 Jun 23;14:656. doi: 10.3389/fnins.2020.00656 (PMC7325604; doi:10.3389/fnins.2020.00656)
Supplement: Supplementary file 3 [file Data_Sheet_1.pdf]

Supplementary Material

1 Supplementary Data

Gross retinal architecture and outer retina

Outer nuclear layer (ONL), neuroblast/inner nuclear layer (NBL/INL) (p5), inner nuclear layer (INL) (p8-p30) and inner plexiform layer (IPL) thickness was quantified (n=4-5; Suppl. Fig. 1) and indicated a significant loss of photoreceptors (ONL) from p14 in all IRD models (Suppl. Fig. 1q). While reduced ONL thickness has been reported in *Rds-/-* mice (Sanyal et al., 1980), ONL thickness akin to wild type was observed in *Rho-/-* (Humphries et al., 1997) and *Tulp1-/-* retinas (Ikeda et al., 2000) at p14. The thickness of NBL/INL differed between IRD models at p5 (Suppl. Fig. 1r) possibly due to differences in timing of transition of NBL/INL into INL. Notably, at p14, the thickness of the IPL (Suppl. Fig. 1s) was significantly reduced in *Tulp1-/-* compared to wt, *Rho-/-* and *Rds-/-* retinas.

Photoreceptor cells

Rod and cone photoreceptors were detected using rhodopsin (RHO; Suppl. Fig. 2) and cone arrestin 3 (ARR3) immunocytochemistries (Suppl. Fig. 3), respectively. RHO was located in the perikarya of developing rod photoreceptors at p5 and p8 and in rod photoreceptor segments of wt retinas by p14 (Suppl. Fig. 2a, 2e, 2i). In contrast, RHO accumulated in the perikarya above wt levels in both *Tulp1-/-* (Suppl. Fig. 2b, 2f, 2j) and *Rds-/-* (Suppl. Fig. 2d, 2h, 2l) by p5 and remained mislocalised at p14; RHO was not detected in *Rho-/-* mice as anticipated (Suppl. Fig. 2c, 2g, 2k). The thickness of the photoreceptor segment layer was significantly reduced in the IRD retinas at p14 (Suppl. Fig. 2).

The majority of cone cell bodies were located at the top of the ONL and were strongly labelled for ARR3 in wt retinas by p8 (Suppl. Fig. 3e, 3i). In contrast, in IRD retinas, cones stained weakly for ARR3 and were dispersed in the forming ONL at p5 (Suppl. Fig 3b, 3c 3d) and p8 (Suppl. Fig. 3f, 3g, 3h). By p14 *Tulp1-/-* retinas (Suppl. Fig. 3j) exhibited similar cone labelling to wt retinas (Suppl. Fig. 3i), while abnormally shaped cones were detected in both *Rho-/-* (Suppl. Fig. 3k) and *Rds-/-* retinas (Suppl. Fig. 3l). Cones were previously reported to be normal in *Rho-/-* (Jaissle et al., 2001) and *Rds-/-* (Travis and Bok, 1993) at p14. In summary, significant perturbations in development of rod and cone photoreceptors between p5 and p14 were observed in all IRD models.

Outer plexiform layer (OPL)

The CTBP2 gene encodes two alternative transcripts producing two distinct proteins; CTBP2 (C-terminal binding protein 2, a transcriptional repressor) and RIBEYE (a major component of the synaptic ribbons in the retina). The CTBP2 antibody used detects both CTBP2 and RIBEYE. Significantly different CTBP2 (RIBEYE) expression was observed in the OPL between IRD and wt retinas (Suppl. Fig. 4). CTBP2 (RIBEYE) expression in the OPL peaked in wt retinas at p8 (Suppl. Fig. 4e) but in *Tulp1-/-* retinas at p14 (Suppl. Fig. 4j). CTBP2 labelling was highest in wt, lower in

Rho-/- and *Rds-/-* and lowest in *Tulp1-/-* at p8 (Suppl. Fig. 4m). Thinning of the CTBP2-positive synaptic region was detected in IRD retinas by p14 (Suppl. Fig. 4j, 4k, 4l). Previously, altered RIBEYE expression was found in the OPL of *Tulp1-/-* mice by p13-p16 (Grossman et al., 2009), however our results suggest deterioration of synaptic terminals begins earlier in *Tulp1-/-* retinas, at least by p8.

Horizontal cells

Horizontal cells were labelled using calbindin 1 (CALB1) antibody (Suppl. Fig. 5). CALB1 labelling of horizontal cell processes in the OPL (Suppl. Fig. 5u) and number of horizontal cells (Suppl. Fig. 5v) were quite similar between wt and IRD retinas. Dendritic arborisation of horizontal cells was considerably reduced in *Tulp1-/-* (Suppl. Fig. 5n) and *Rds-/-* retinas (Suppl. Fig. 5p) by p14. Ectopic sprouting of horizontal cell neurites started in *Rds-/-* (Suppl. Fig. 5h, 5l) and *Rho-/-* (Suppl. Fig. 5g, 5k) retinas at p8 and was widespread in all IRD models at p14 (Suppl. Fig. 5n, 5o, 5p, 5r, 5s, 5t). The observed ectopic sprouting of horizontal cells (along with decreased expression of CTPB2) suggests a breakdown of synaptic connectivity and remodelling in the INL of these IRD retinas by p8-p14.

2 Supplemental Figures

Suppl. Fig. 1.

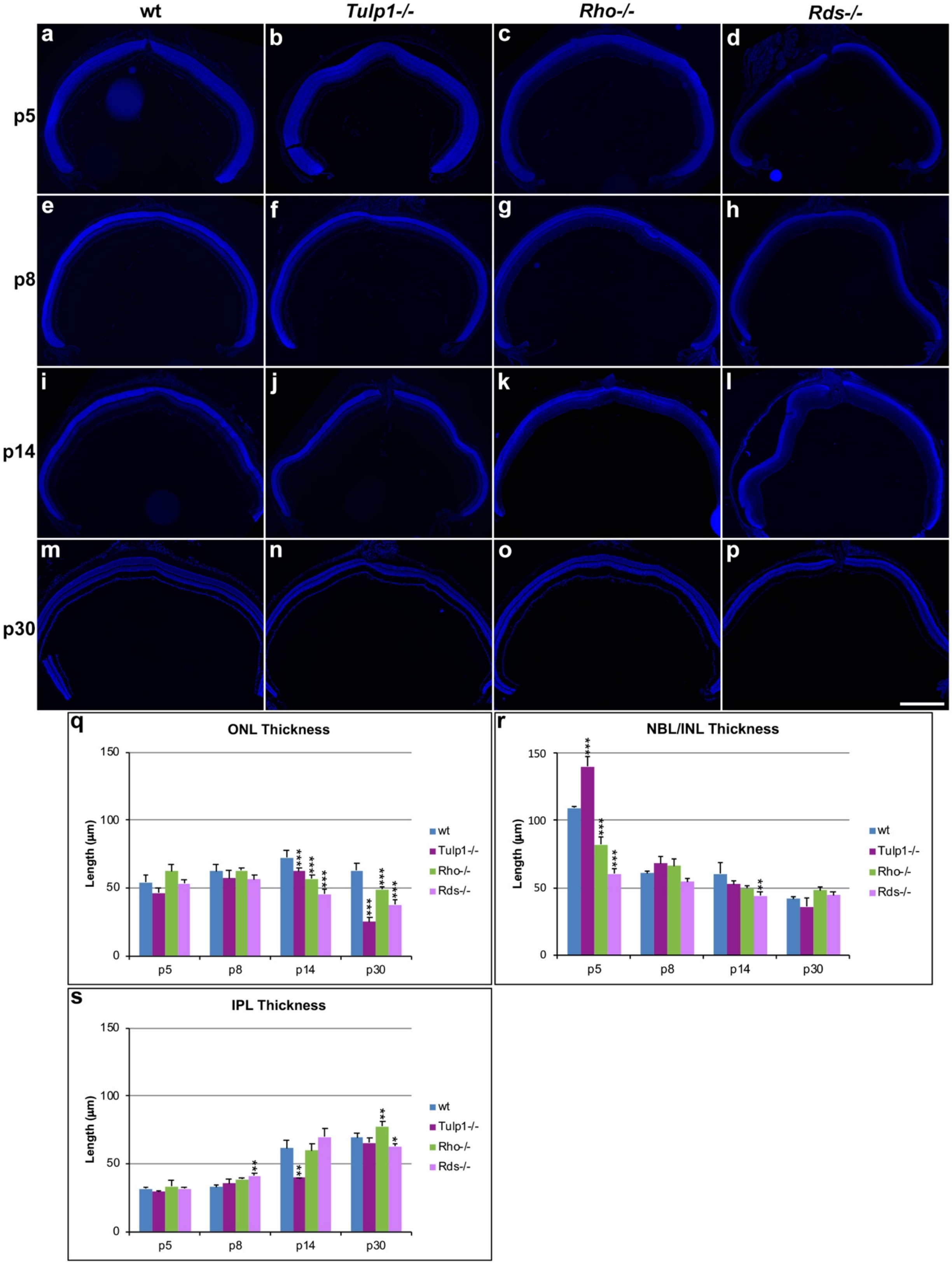

Suppl. Fig. 1. Gross retinal architecture

Retinal sections from wt, *Tulp1-/-*, *Rho-/-* and *Rds-/-* mice at p5, p8, p14 and p30 were counterstained with DAPI (blue). **a-p**: Lateral frames of microscope images were stitched together to create pan-retinal images in cellSens software. The thickness of the outer nuclear layer (ONL; **q**), neuroblast/inner nuclear layer (NBL/INL; **r**) and inner plexiform layer (IPL; **s**) were measured in the central retina (in sections within 200 µm of the optic nerve) using cellSens (n=4-5) and results are given in bar charts; bars represent mean+SD. Scale bar (**p**): 500 µm. *: $p<0.05$ **; $p<0.01$; ***: $p<0.001$; p values refer to differences between IRD and wt mice (ANOVA).

Suppl. Fig 2.

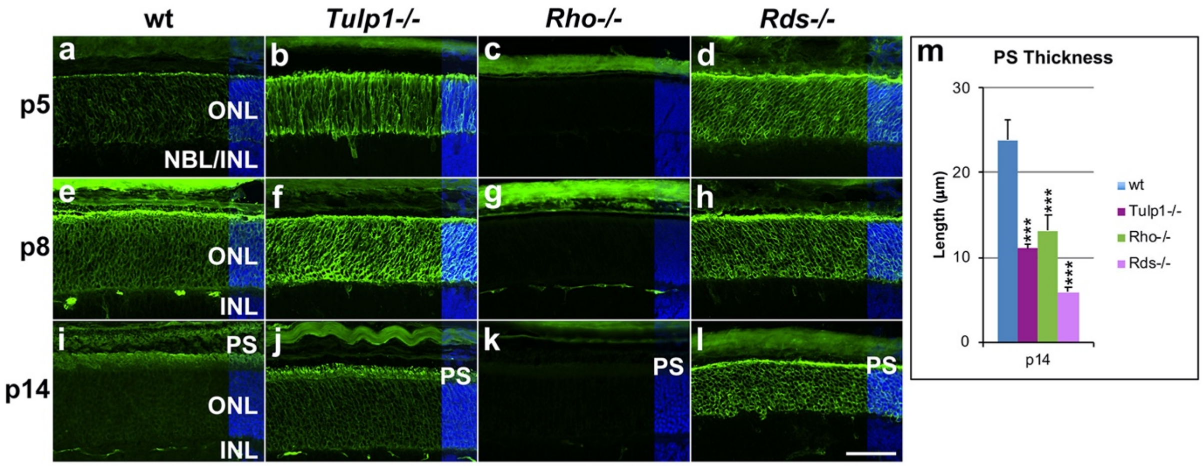

Suppl. Fig. 2. Rod photoreceptors assessed by RHO immunocytochemistry

Retinas from wt, *Tulp1-/-*, *Rho-/-* and *Rds-/-* mice were evaluated at p5, p8 and p14 (n=5-6). **a-l**: Sections were labelled with RHO immunocytochemistry (green) and counterstained with DAPI (blue; overlaid on the right). **m**: Thickness of the photoreceptor segment layer (PS) is given in a bar chart; bars represent mean+SD. ONL: outer nuclear layer, INL: inner nuclear layer, NBL: neuroblast layer. Scale bar (**l**): 50 µm. ***: p<0.001 between IRD and wt retinas (ANOVA).

Suppl. Fig. 3.

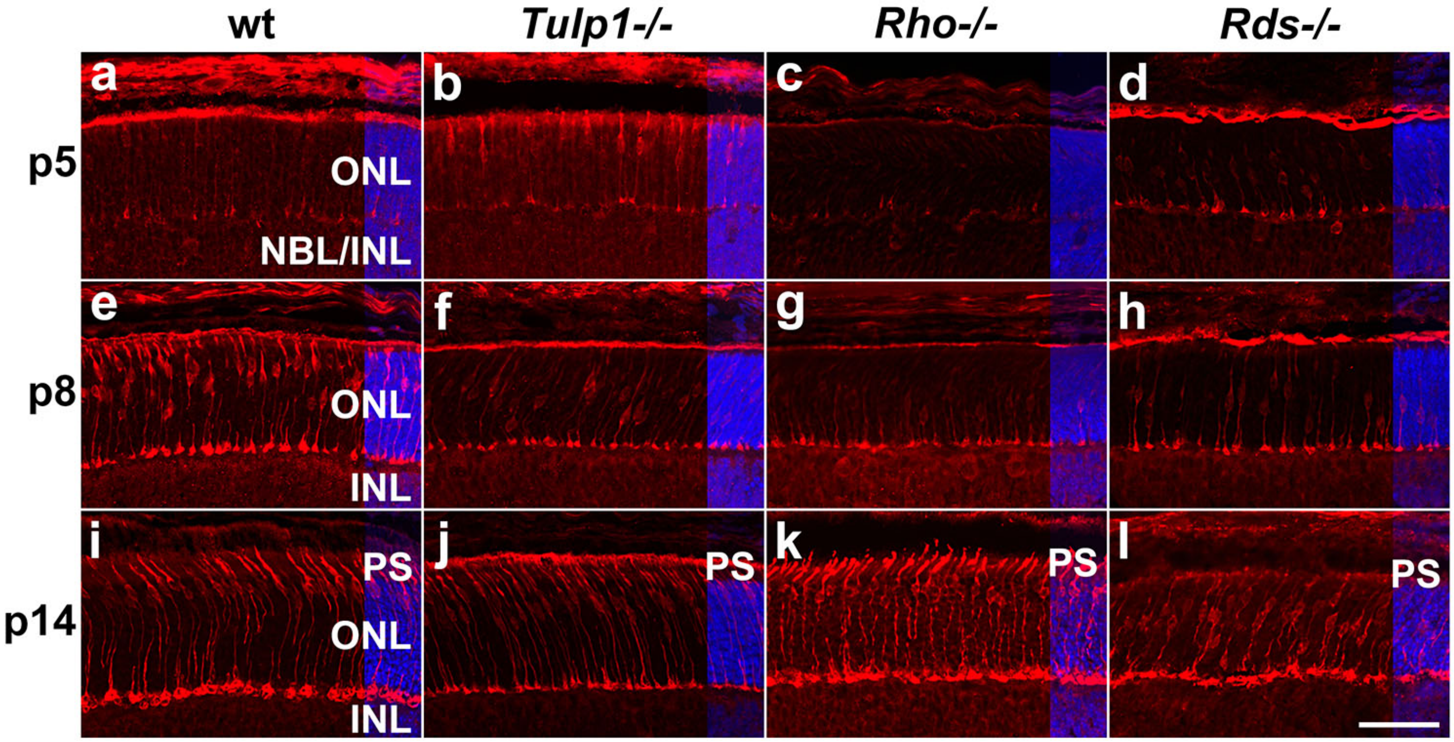

Suppl Fig. 3. Cone photoreceptors visualised by ARR3 immunocytochemistry

Retinas from wt, *Tulp1-/-*, *Rho-/-* and *Rds-/-* mice were evaluated at p5, p8 and p14 (n=5-6). Sections were labelled with ARR3 immunocytochemistry (red) and counterstained with DAPI (blue; overlaid on the right). ONL: outer nuclear layer, INL: inner nuclear layer, NBL: neuroblast layer. Scale bar (**l**): 50 µm.

Suppl. Fig. 4.

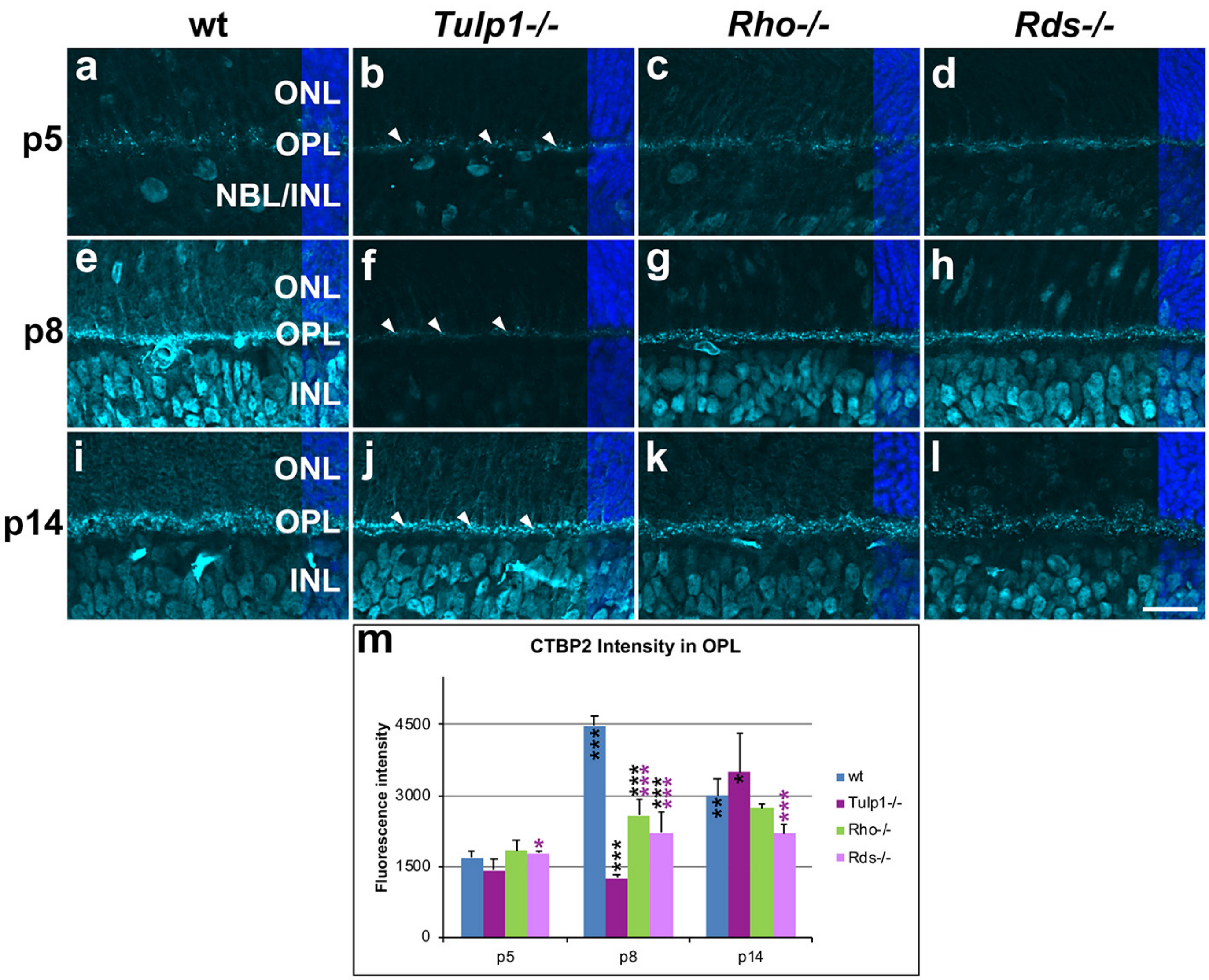

Suppl. Fig. 4. CTBP2 (RYBEYE) immunocytochemistry in the OPL

Retinas from wt, *Tulp1-/-*, *Rho-/-* and *Rds-/-* mice were taken at p5, p8 and p14 (n=5-6). Sections were labelled with CTBP2 (A-L; light blue) immunocytochemistry and counterstained with DAPI (dark blue; overlaid on the right). Q: CTBP2 (RYBEYE) label intensities in the OPL were measured in microscope images using cellSens software (n=4) and results are given in a bar chart; bars represent mean+SD. ONL: outer nuclear layer, OPL: outer plexiform layer, INL: inner nuclear layer, NBL: neuroblast layer. Scale bar (**l**): 20 µm. Arrowheads indicate the OPL in *Tulp1-/-* sections. *: $p<0.05$; **: $p<0.01$; ***: $p<0.001$ (ANOVA). Black stars above bars refer to differences between IRD and wt mice, purple stars above bars refer to differences between *Tulp1-/-* and the other IRD mice; black stars within bars refer to a difference compared to the previous time point.

Suppl. Fig. 5.

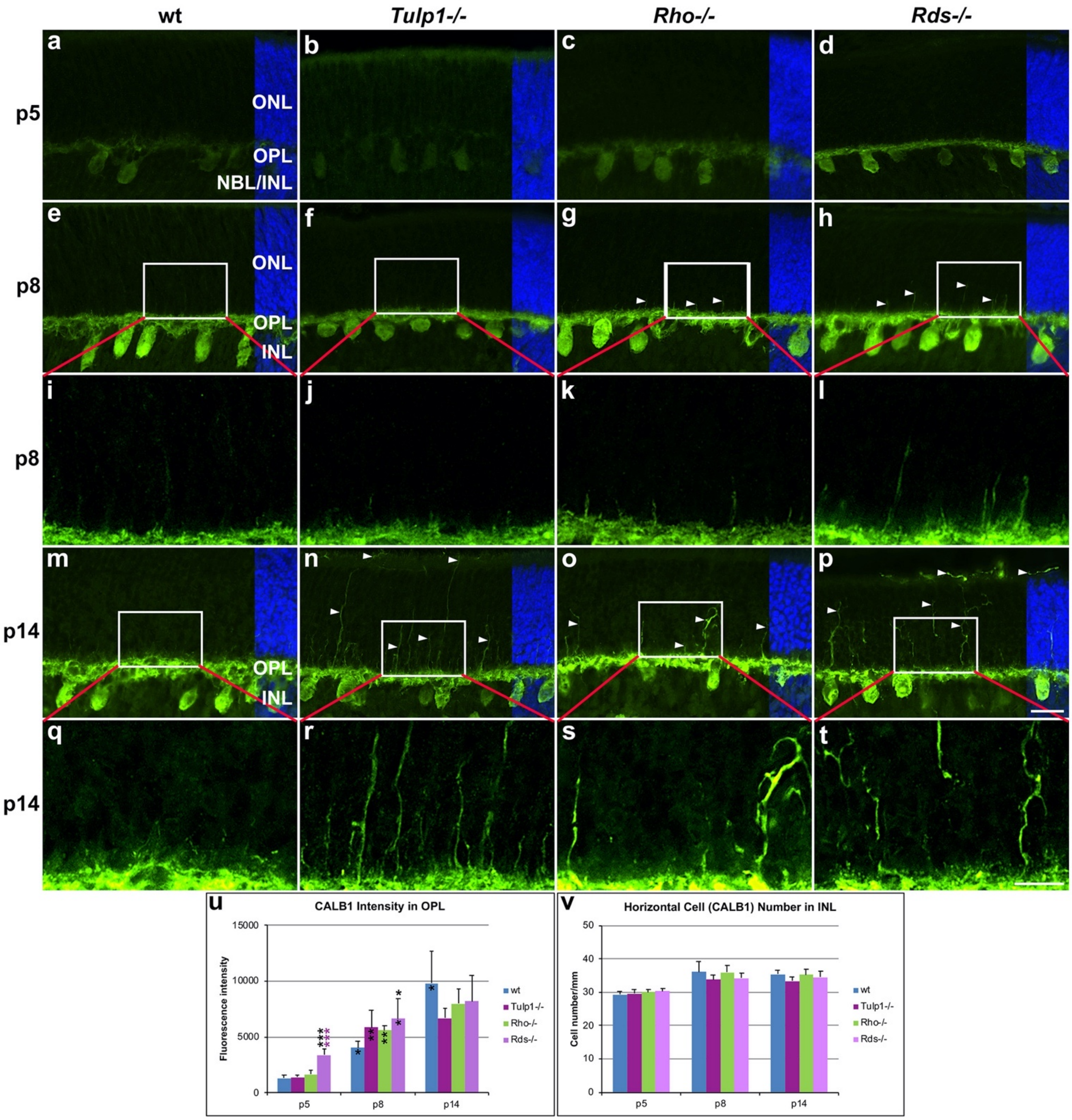

Suppl. Fig. 5. Horizontal cells evaluated using CALB1 immunocytochemistry in the OPL/INL region

Retinas from wt, *Tulp1-/-*, *Rho-/-* and *Rds-/-* mice were taken at p5, p8 and p14 (n=5-6). Sections were labelled with CALB1 immunocytochemistry (green) and counterstained with DAPI (blue; overlaid on the right). CALB1 label intensities in the OPL (**u**) and CALB1-positive cell numbers in the INL (**v**) were quantified in microscope images using cellSens software (n=4) and results are given in bar charts;

bars represent mean+SD. Inserts on **e**, **f**, **g** and **h** are magnified on **i**, **j**, **k** and **l**, respectively, while insert on **m**, **n**, **o** and **p** are magnified on **q**, **r**, **s** and **t**, respectively. ONL: outer nuclear layer, OPL: outer plexiform layer, INL: inner nuclear layer, NBL: neuroblast layer. Arrowheads indicate ectopic neurites of horizontal cells in the ONL. Scale bar (**p**): 20 µm. Scale bar (**t**): 10 µm. *: p<0.05; **: p<0.01; ***: p<0.001 (ANOVA). Black stars above bars refer to differences between IRD and wt mice, purple stars above bars refer to differences between *Tulp1-/-* and the other IRD mice; black stars within bars refer to a difference compared to the previous time point.

Suppl. Fig. 6.

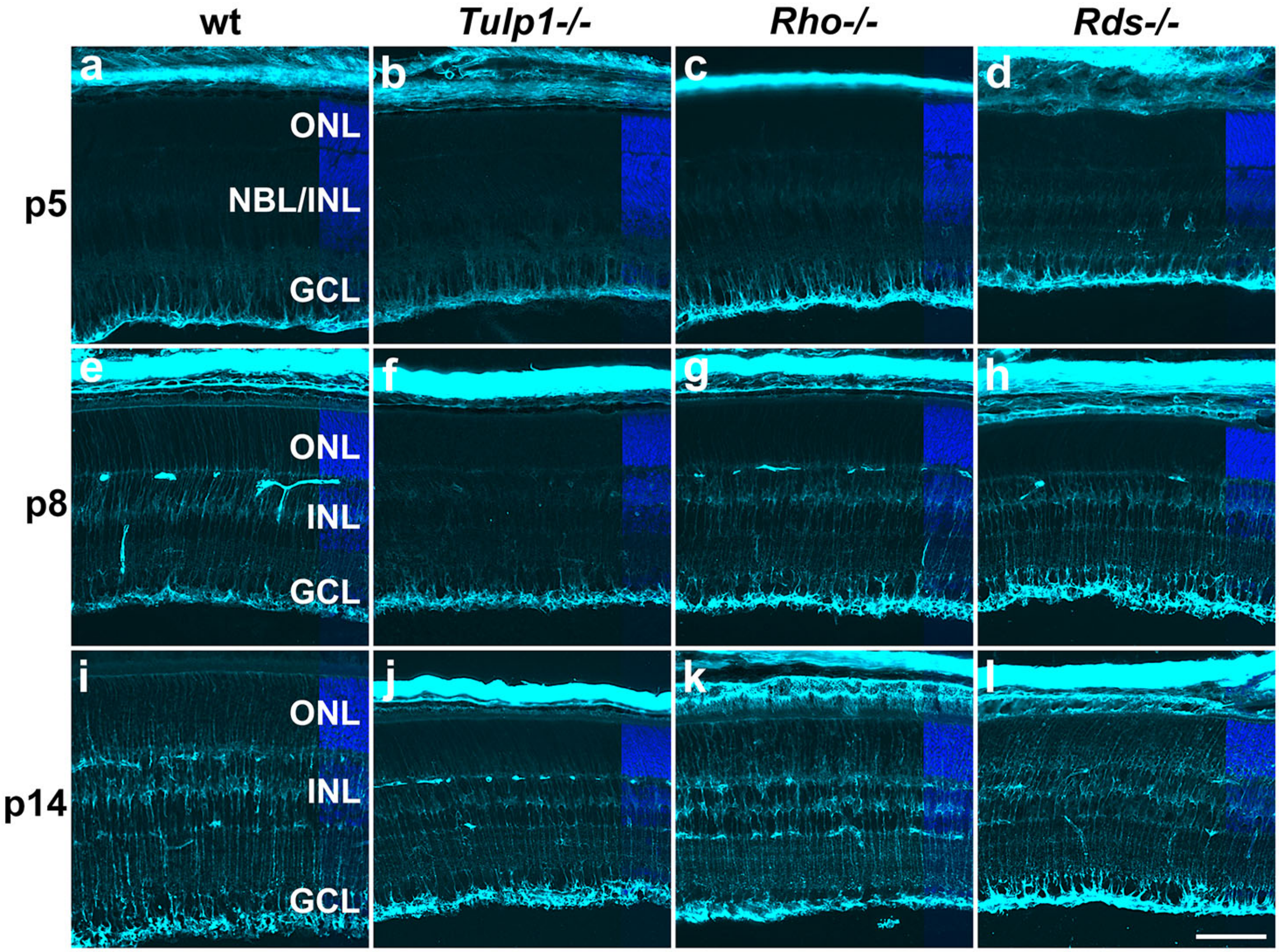

Suppl. Fig. 6. Muller cells labelled using CRALBP immunocytochemistry

Retinas from wt, *Tulp1-/-*, *Rho-/-* and *Rds-/-* mice were taken at p5, p8 and p14 (n=5-6). Sections were labelled with CRALBP immunocytochemistry (light blue) and counterstained with DAPI (dark blue; overlaid on the right). NBL: neuroblast layer, ONL: outer nuclear layer, INL: inner nuclear layer, GCL: ganglion cell layer. Scale bar (**l**): 50 μm.

Suppl. Fig. 7.

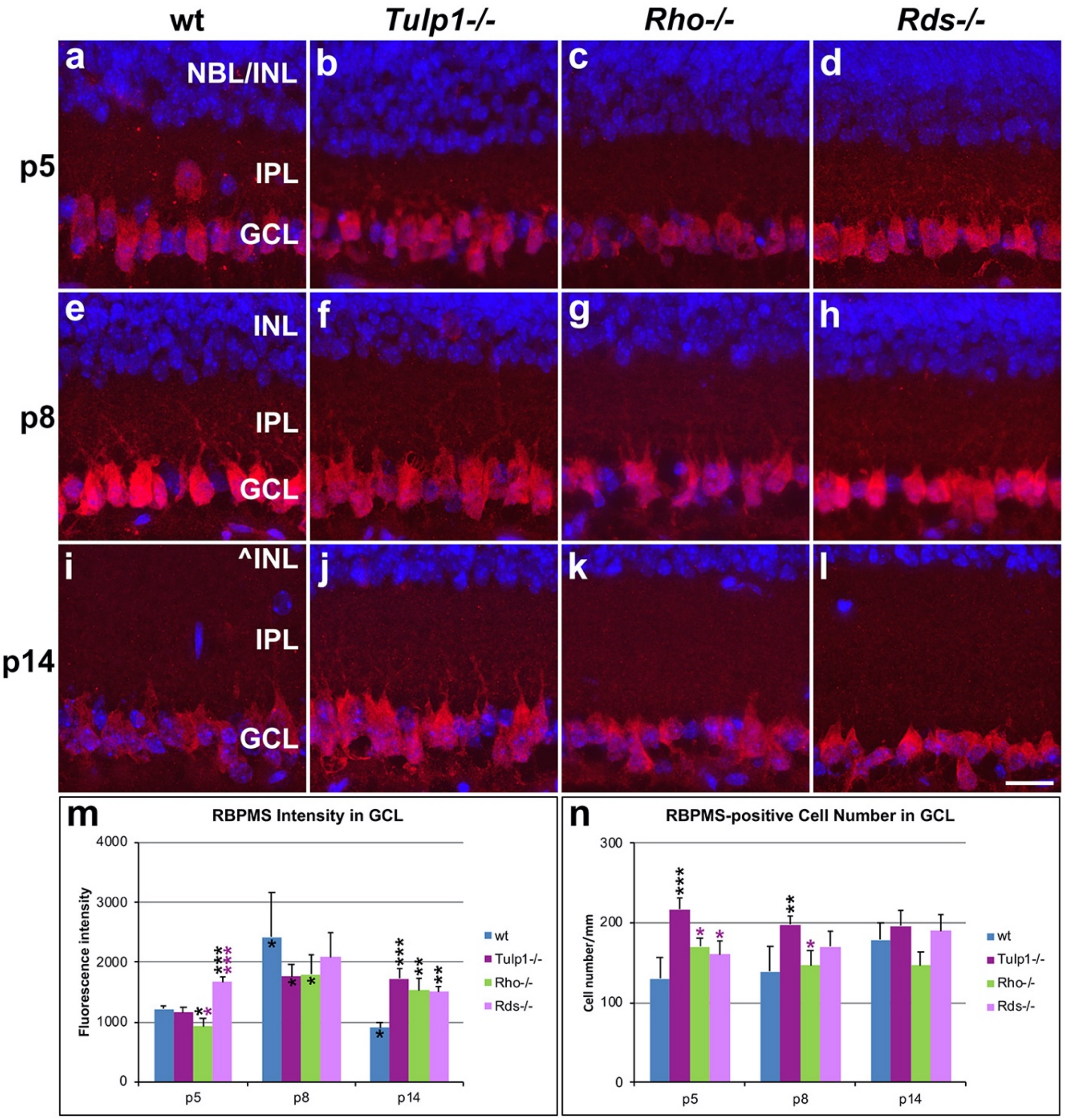

Suppl. Fig. 7. Ganglion cells labelled using RBPMS immunocytochmistry

Retinas from wt, *Tulp1-/-*, *Rho-/-* and *Rds-/-* mice were taken at p5, p8 and p14 (n=5-6). **a**-**l**: Sections were labelled with RBPMS immunocytochemistry (red) and counterstained with DAPI (blue). RBPMS label intensities (n=4; **m**) and RBPMS-positive cell numbers (n=4; **n**) in the ganglion cell layer (GCL) were quantified from microscope images using cellSens software and results are given in bar charts; bars represent mean+SD. INL: inner nuclear layer, NBL: neuroblast layer. Scale bar (**l**): 20 µm. *: $p<0.05$ **; $p<0.01$; ***: $p<0.001$ (ANOVA). Black stars above bars refer to differences between IRD and wt mice, purple stars above bars refer to differences between *Tulp1-/-* and the other IRD mice; black stars within bars refer to a difference compared to the previous time point.

Suppl. Fig. 8.

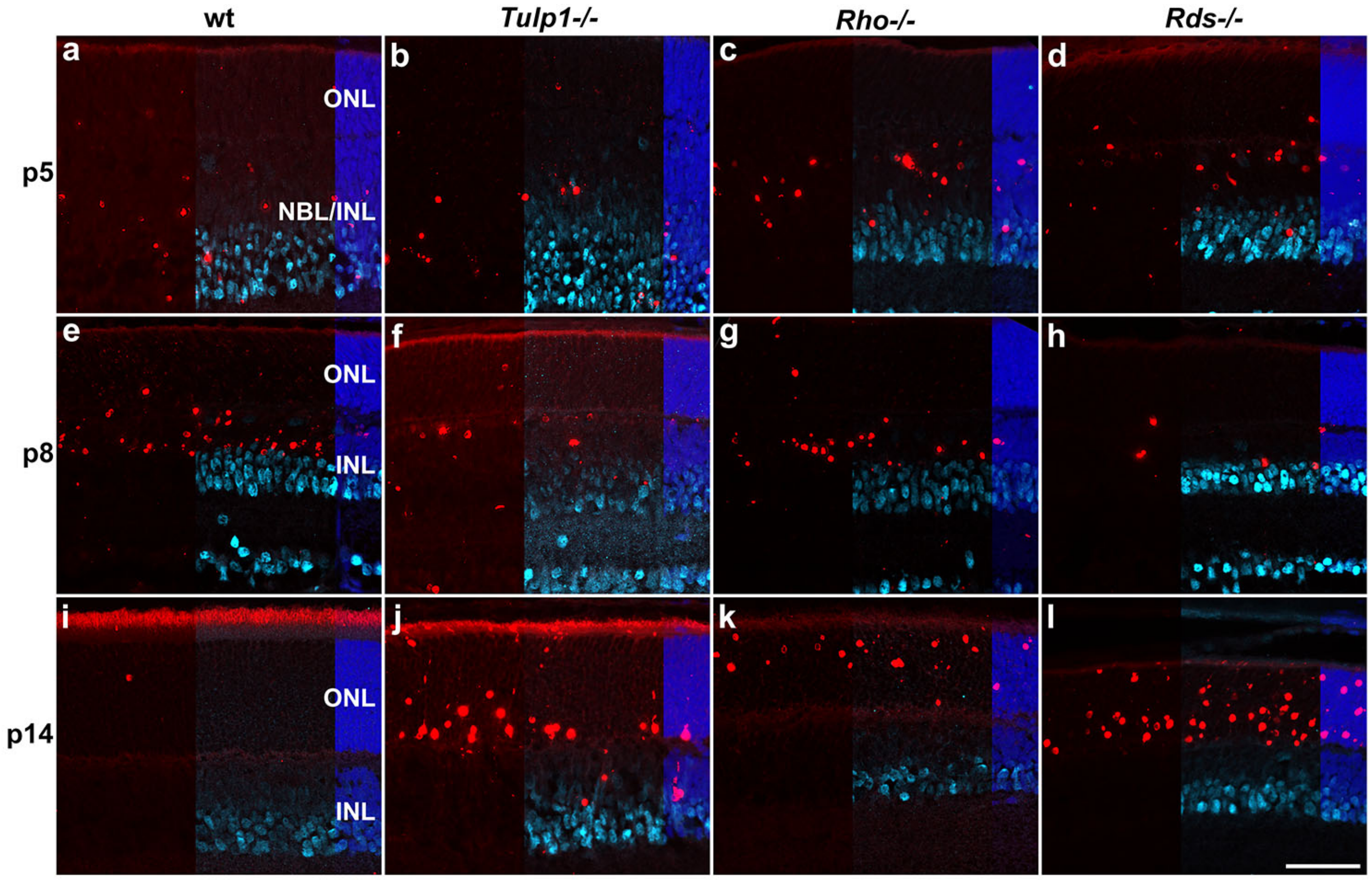

Suppl. Fig. 8. Combined TUNEL stain and PAX6 immunocytochemistry in the ONL – INL region

Retinas from wt, *Tulp1-/-*, *Rho-/-* and *Rds-/-* mice were taken at p5, p8 and p14 (n=3). Sections were co-labelled with TUNEL stain (red) and PAX6 immunocytochemistry (light blue and overlaid on the right side) and counterstained with DAPI (dark blue, overlaid on the far right). ONL: outer nuclear layer, INL: inner nuclear layer, GCL: ganglion cell layer, NBL: neuroblast layer. Scale bar (**l**): 50 µm.

3 Supplemental data given in separate files

Suppl. Fig. 9. Zoom-enabled version of Fig. 9 - Expression of a high-confidence TULP1 interactome genes in various retinal cell types

A list of 33 TULP1 interactors were assembled from predicted (by both PrePPI and STRING) and 11 validated interactors. Expression of these genes in various retinal cell types was determined from Gene Expression Omnibus data sets; GSE97534, GSE59201, GSE115404, GSE127771, GSE86199, GSE19304, and is given in row-scaled clustered heatmap representation in some p4-p7 (**a**) and adult (**b**) retinal cell types and in developing cone cells at p2, p5, p8, p12 and adult (**c**). Note that in panel (**a**), rod, cone and ganglion cells are from p5, bipolar cells are from p7 and Muller cells are from p4 retinas. pr: photoreceptor.

Suppl. Fig 10. Expression of a medium-confidence TULP1 interactome genes in various retinal cell types (zoom-enabled)

A list of 229 TULP1 interactors were assembled from PrePPI- and STRING-predicted, and 11 validated interactors. Expression of these genes in various retinal cell types was determined from Gene Expression Omnibus data sets; GSE97534, GSE59201, GSE115404, GSE127771, GSE86199, GSE19304. The 20% highest expressed interactor genes (46) from each selected cell type were analysed in p4-p7 (**a**, **b**, **c**), and adult (**d**, **e**, **f**) retinal cell types, and in developing cones (**g**, **h**, **i**). **a**, **d**, **g**: Row-scaled clustered heatmap representation of the interactor genes; **b**, **e**, **h**: Venn diagram representation of the interactor genes; **c**, **f**, **i**: Cell type specific expression of the interactor genes.

Suppl. Fig. 11. Zoom-enabled version of Fig 10 - Cell type-specific expression of IRD genes in mouse retina at p4-p7

Retinal cell type-specific gene expression values of 235 mouse orthologues of a panel of 251 human IRD genes (Dockery et al., 2017) were determined using GSE59201, GSE97534, GSE71462, GSE33088, GSE127771, GSE86199, GSE115404 data sets from Gene Expression Omnibus. Photoreceptor contamination of non-photoreceptor samples was determined utilising *Rho* expression and corresponding photoreceptor derived IRD gene expression values deducted from these samples. Expression values are given in a clustered heatmap format; pr: photoreceptor. A zoom-enabled version of this figure is given as Suppl. Fig. 11.

Suppl. Table 1. TULP1 interactors

Suppl Table 2. List of 251 IRD genes (Dockery et al. 2017)
