# Supplementary figures and images for: Non-photoreceptor Expression of Tulp1 May Contribute to Extensive Retinal Degeneration in Tulp1-/- Mice
Source: Front Neurosci. 2020 Jun 23;14:656. doi: 10.3389/fnins.2020.00656 (PMC7325604; doi:10.3389/fnins.2020.00656)

**a. Retinal Cell Types at p4-p7**

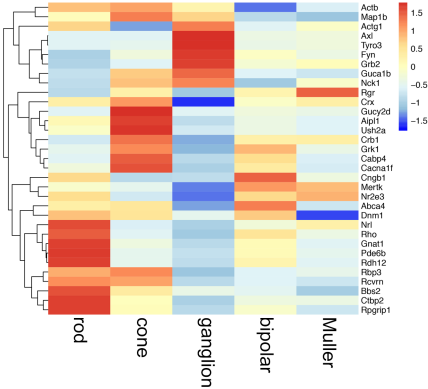

**b. Adult Retinal Cell Types**

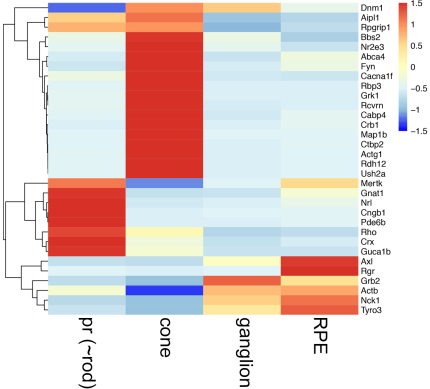

**c. Cone Development**

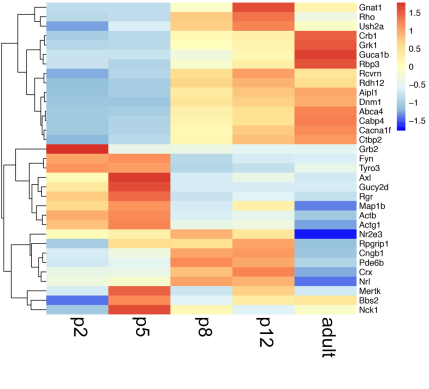

Supplement: Supplementary file 4 [file Image_1.PDF]

## Retinal Cell Types at p4-p7

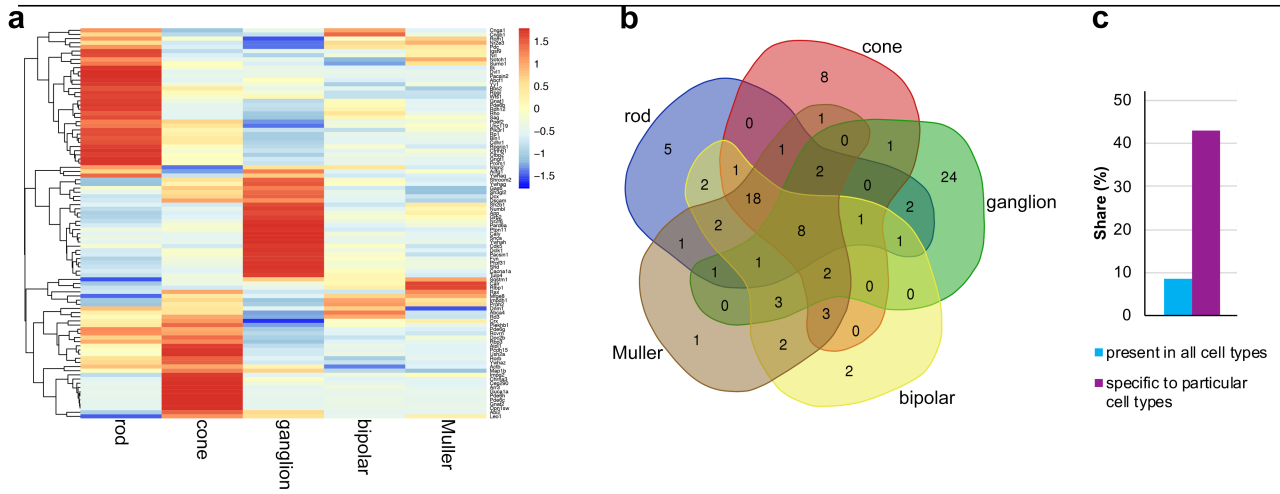

## Adult Retinal Cell Types

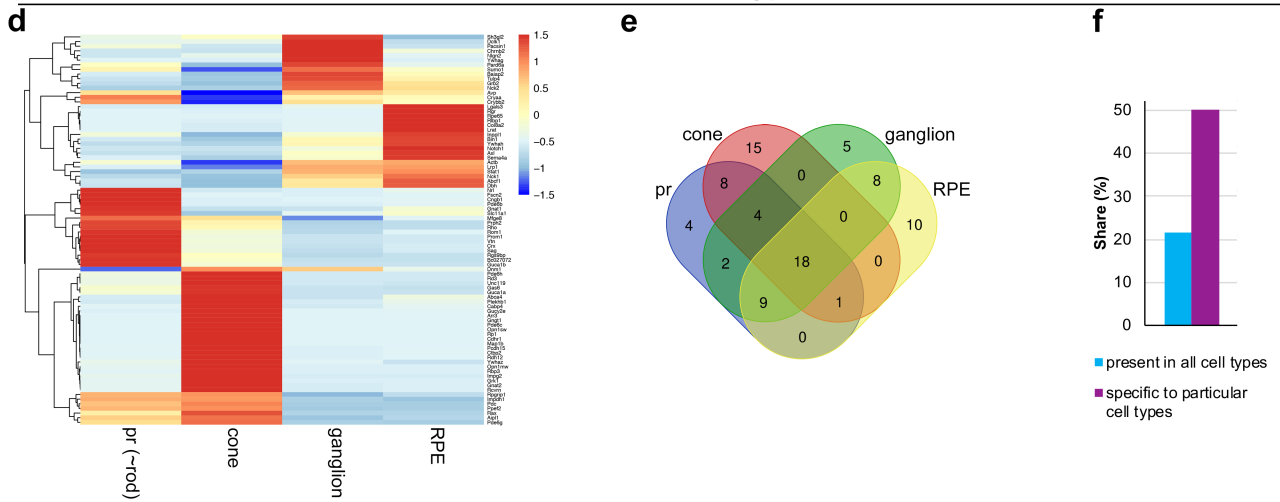

## Cone Development

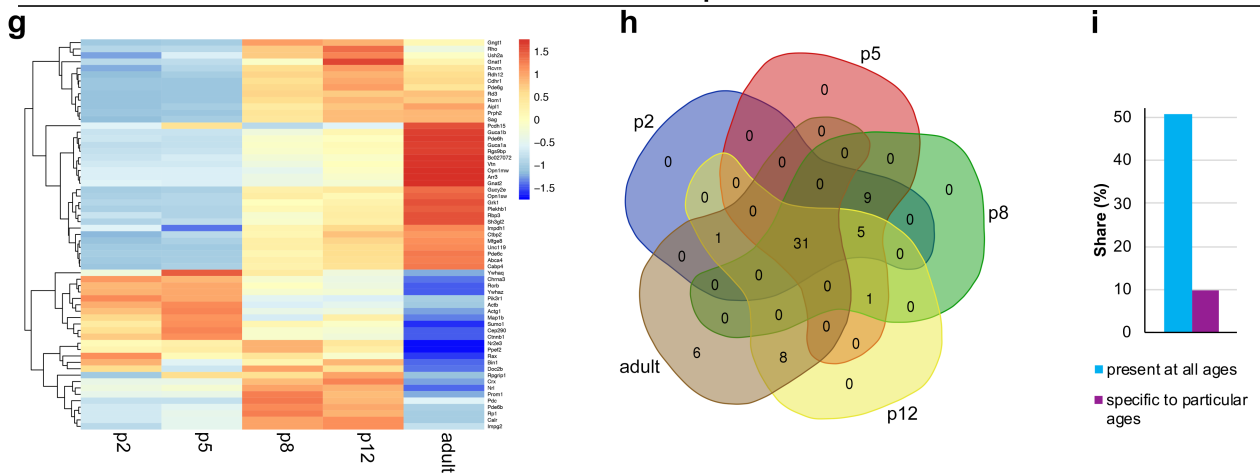

Supplement: Supplementary file 5 [file Image_2.PDF]

IRD genes (weighted merged photoreceptors)

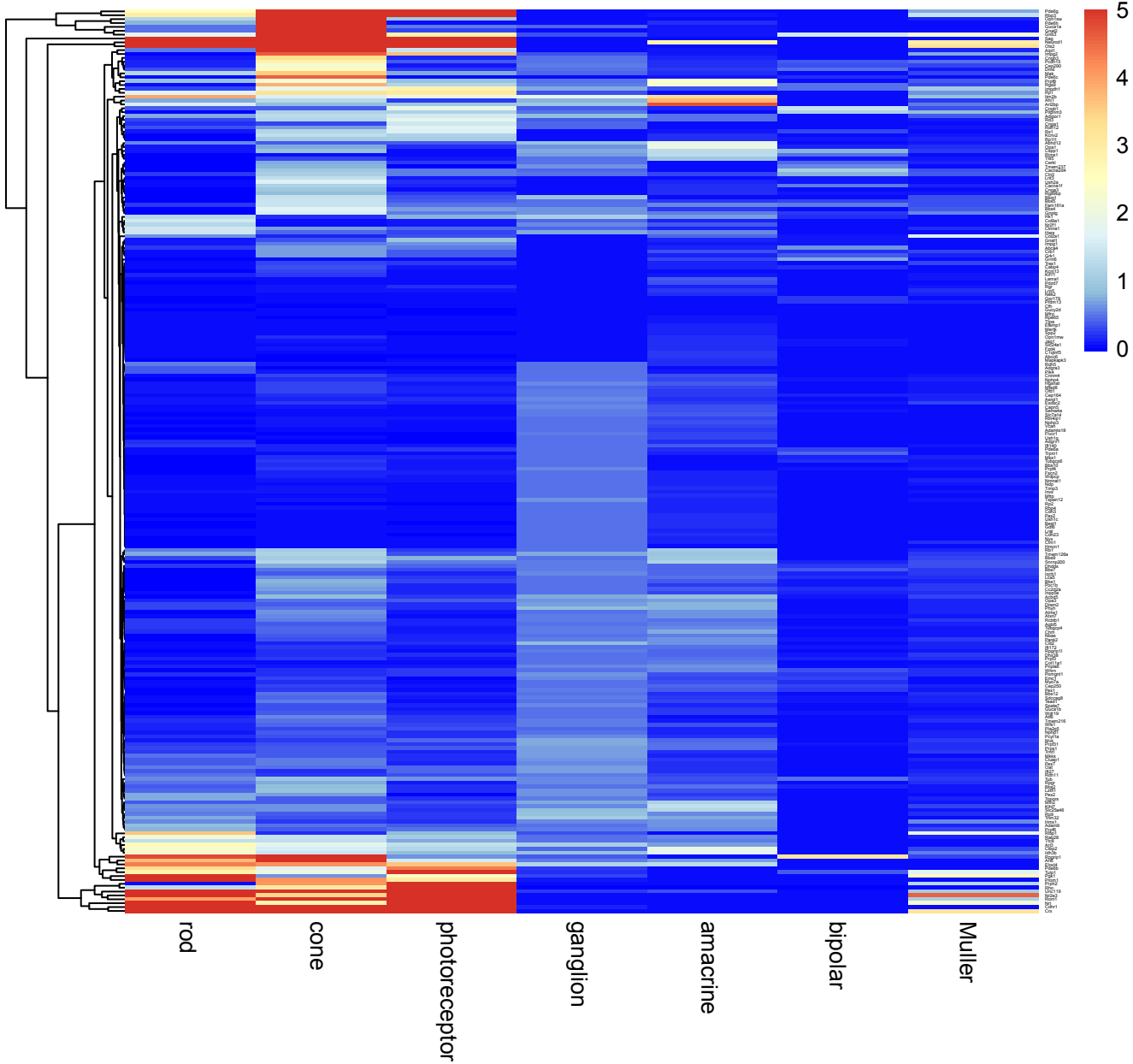

Supplement: Supplementary file 6 [file Image_3.PDF]
